# Flexural Crack Bridging Across Loading Stages in Hybrid Steel Fiber Mortar Prepared with a Magnetic Field: A Model of Orientation and Dispersion

**DOI:** 10.3390/ma19214588

**Published:** 2026-10-29

**Authors:** Yuchen Zhang, Xiulei Li, Xiaoyan Yang, Xun Wu

**Affiliations:** 1 State Key Laboratory of Disaster Prevention & Mitigation of Explosion & Impact, Army Engineering University of PLA, Nanjing 210007, China; 2 State Key Laboratory of Water Disaster Prevention, Hohai University, Nanjing 210024, China; 3 Key Laboratory of Ministry of Education for Geomechanics and Embankment Engineering, Hohai University, Nanjing 210024, China; 4 Key Laboratory of Geological Hazards Mitigation for Mountainous Highway and Waterway, Chongqing Jiaotong University, Chongqing 400074, China; 5 School of Civil Engineering, Chongqing Jiaotong University, Chongqing 400074, China

**Keywords:** hybrid steel fiber mortar, magnetic field-assisted preparation, flexural crack bridging, fiber pullout, model of orientation and dispersion

## Abstract

Fiber scale, dosage, and preparation govern flexural crack bridging in mortar reinforced with steel fibers. Straight fine steel fibers (FSF, 16 mm) and common steel fibers (CSF, 30 mm) were evaluated by central point flexural testing of mortars containing a single fiber type and 1.60 vol.% hybrid mortars prepared through magnetic field treatment (A) and vibration (R). Corrected nominal stress, residual stress at common machine displacements, work partitioned by loading stage, digital image correlation (DIC), fracture surface observations, scanning electron microscopy (SEM), and a multiscale bridging model of orientation and dispersion were combined. Among the tested contents, performance with a single fiber type peaked at 0.80% FSF and 1.60% CSF. The A route increased peak stress by 15.3–45.3% in mixtures containing a single fiber type and 18.7–34.9% in hybrids, with larger gains in late stress and cumulative energy. The CSF:FSF = 2:1 A mixture reached a peak stress of 26.26 MPa and cumulative flexural energy of 17.06 N·m, exceeding baselines calculated using the rule of mixtures under matched preparation conditions by 40.2% and 60.9%, respectively. Responses at common displacements and qualitative image observations supported stronger early bridging by FSF and more persistent bridging by CSF at large crack openings. The model connects effective fiber populations, single-fiber forces, bridging traction, and sectional response to explain the contributions of the two fiber scales during loading. These results reveal complementarity between FSF and CSF across loading stages and interactions that vary with composition.

## 1. Introduction

Flexural fracture in cementitious materials progresses through matrix deformation, crack initiation, strain localization, opening of a dominant crack, and residual load transfer. Steel fibers modify this progression by transmitting tensile forces across active cracks. At small crack openings, the response is controlled by elastic load transfer and interfacial debonding; continued opening activates frictional sliding and fiber pullout, which sustain residual resistance and dissipate energy [1,2,3]. Fiber inclination, geometry, matrix composition, and anchorage length govern the mobilized pullout force and its evolution with crack opening [4,5,6,7]. These micromechanical processes determine different portions of the structural response, so crack initiation stress, peak resistance, residual stress, and accumulated flexural energy represent quantities associated with distinct loading stages [8,9].

Fiber volume fraction defines the potential reinforcement population, while mixing, flow, casting, and confinement determine the fibers that effectively intersect a developing crack. Vibration combined with controlled steel fiber orientation has also improved the flexural performance of masonry mortars [10]. Flowability and placement conditions can create preferential orientation, wall effects, and spatially heterogeneous fiber populations [11,12,13]. Mortar rheology also controls fiber rotation and translation during casting, linking fresh mortar behavior to the final arrangement of fibers crossing cracks [14,15,16]. Increasing fiber content raises the potential number of bridges and can also increase fiber interaction, disturb flow, and reduce placement efficiency [17,18]. The resulting mechanical response may reach a maximum within the tested dosage range and decline at a higher content. Fiber dosage, effective orientation, and spatial activation therefore represent separate variables.

Hybrid reinforcement introduces more than one bridging length scale. Short fine fibers provide a high potential number density and can intercept distributed discontinuities during crack initiation and early localization [19,20,21]. Longer fibers develop greater anchorage lengths and remain active as a dominant crack reaches larger openings [22,23,24,25]. Previous studies have reported improved flexural resistance and energy absorption through fiber hybridization, while the magnitude of the improvement depends on fiber dimensions, total dosage, composition, and the selected response metric [26,27,28,29]. Tests on dry UHPC also show that the effects of combining steel fiber lengths vary among flexural strength, fracture toughness, and fracture energy [30]. Direct ranking of hybrid mixtures identifies the strongest tested composition. Comparison with the corresponding systems containing a single fiber type determines how the hybrid response relates to the expected component contributions. A baseline calculated using the rule of mixtures under matched preparation conditions quantifies hybrid synergy and its development during loading.

Preparation assisted by a magnetic field provides an active route for modifying the arrangement of ferromagnetic steel fibers before matrix setting [31]. Previous studies have shown that electromagnetic treatment can rotate steel fibers toward the nominal field direction and increase flexural strength, resistance after cracking, or energy absorption [32,33,34,35]. The achieved fiber state depends on field intensity, treatment duration, mortar rheology, fiber geometry, aggregate obstruction, and specimen position within the field [36,37,38,39]. Applications have expanded from mortar specimens to fracture testing and reinforced ultrahigh-performance concrete members [40,41]. Most available investigations focus on one fiber scale or characterize performance through peak strength and total toughness. Hybrid systems motivate evaluation of how treatment gains evolve from crack initiation to fiber pullout at large crack openings.

A mechanistic model connects mixture composition to the measured flexural response across several scales. Fiber volume and geometry define the potential population; orientation and placement determine effective crack crossings; embedment and interface properties govern the force carried by an individual fiber; and the resulting traction on the crack face supplies an additional sectional moment. Existing formulations describe flexural response, fiber pullout, and softening as a function of orientation [42,43,44,45]. Orientation measurements from sections and placement models also provide tools for characterizing effective fiber distributions [46,47]. Parameters are identified from the mean responses of systems containing a single fiber type. The framework is used to interpret the contributions of effective fiber populations, orientation and activation, dispersion, and single-fiber pullout during loading.

DIC provides spatial evidence of strain localization and crack path development during flexural loading [48,49,50]. Fracture surfaces and SEM provide complementary observations of exposed fibers, attached mortar, interface separation, local cracking, and apparent pullout paths [3,8]. Calibrated image scale, correlation settings, standardized sections, and synchronized records of load and images support quantitative interpretation [51,52]. In this study, image evidence is used for qualitative assessment of plausible mechanisms.

This study investigates flexural crack bridging across loading stages in mortar reinforced with straight, smooth FSF and CSF, with nominal lengths of 16 and 30 mm. The experimental matrix includes three FSF contents, three CSF contents, and four hybrid compositions at a total fiber content of 1.60 vol.%, with CSF:FSF ratios of 1:2, 1:1, 2:1, and 3:1. Each fiber composition was produced under paired A and R conditions. These labels identify preparation conditions.

Central point flexural responses are evaluated using corrected nominal stress, residual stress at common machine displacements, work partitioned by loading stage, the gain of A relative to R, and departures from baselines for a single fiber type under matched preparation conditions. Qualitative DIC fields, fracture surfaces, and SEM morphology are used to examine crack localization and interface processes. A multiscale bridging model of orientation and dispersion is calibrated on the mean responses of groups containing a single fiber type to explain stage-dependent bridging. The study addresses three questions: how fiber scale changes the active bridging stage and the highest response among the tested dosages; how preparation assisted by a magnetic field affects initiation, peak, and response at large crack openings; and how the complementary contributions of the two fiber scales relate to composition-dependent flexural resistance and energy absorption.

## 2. Materials and Methods

### 2.1. Raw Materials and Steel Fibers

The mortar comprised PO 42.5R cement, quartz sand, and water. The cement had a specific surface area of 368 m^2^ kg^−1^. The sand particle size ranged from 0.063 to 2.0 mm, with a fineness modulus of 2.6.

**Figure 1:**
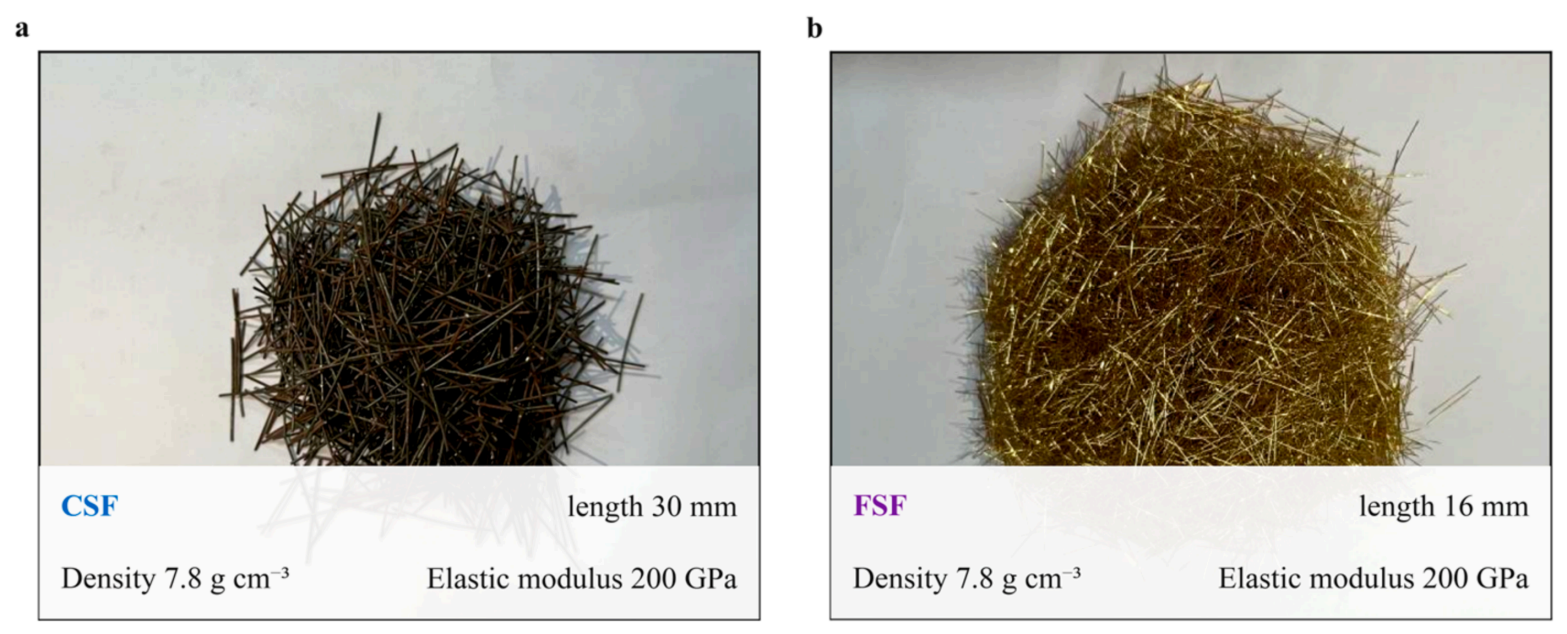
Steel fiber appearance, nominal lengths, density, and elastic modulus. (**a**) CSF; (**b**) FSF.

FSF and CSF were straight and smooth with blunt rounded ends, nominal lengths of 16 and 30 mm, a density of 7.8 g cm^−3^, and an elastic modulus of 200 GPa (Figure 1). Model diameters of 0.20 and 0.55 mm were adopted with reference to [53,54], and tensile strength caps were set to 1800 and 1200 MPa, respectively. Dosage is expressed as mortar volume fraction.

### 2.2. Mixture Design and Specimen Preparation

The cement, sand, and water contents were 585, 1758, and 263 kg m^−3^, respectively, giving a water-to-cement ratio of 0.450. The experimental matrix comprised plain mortar (PC), three dosages each of FSF and CSF, and four hybrid compositions at a total fiber content of 1.60 vol.% (Table 1).

Cement and sand were dry-mixed for 30 s, followed by mixing with water for 60 s. Steel fibers were added gradually, followed by a further 60 s of mixing. The mortar was placed in 40 × 40 × 160 mm molds and vibrated for 60 s, with the coil energized for A and switched off for R. The specimens were covered with film and stored in the molds for 24 h, then demolded and cured in water at 20 ± 2 °C until 28 d after casting. Three specimens were prepared for each composition under each preparation condition.

### 2.3. Magnetic Field-Assisted Preparation

**Figure 2:**
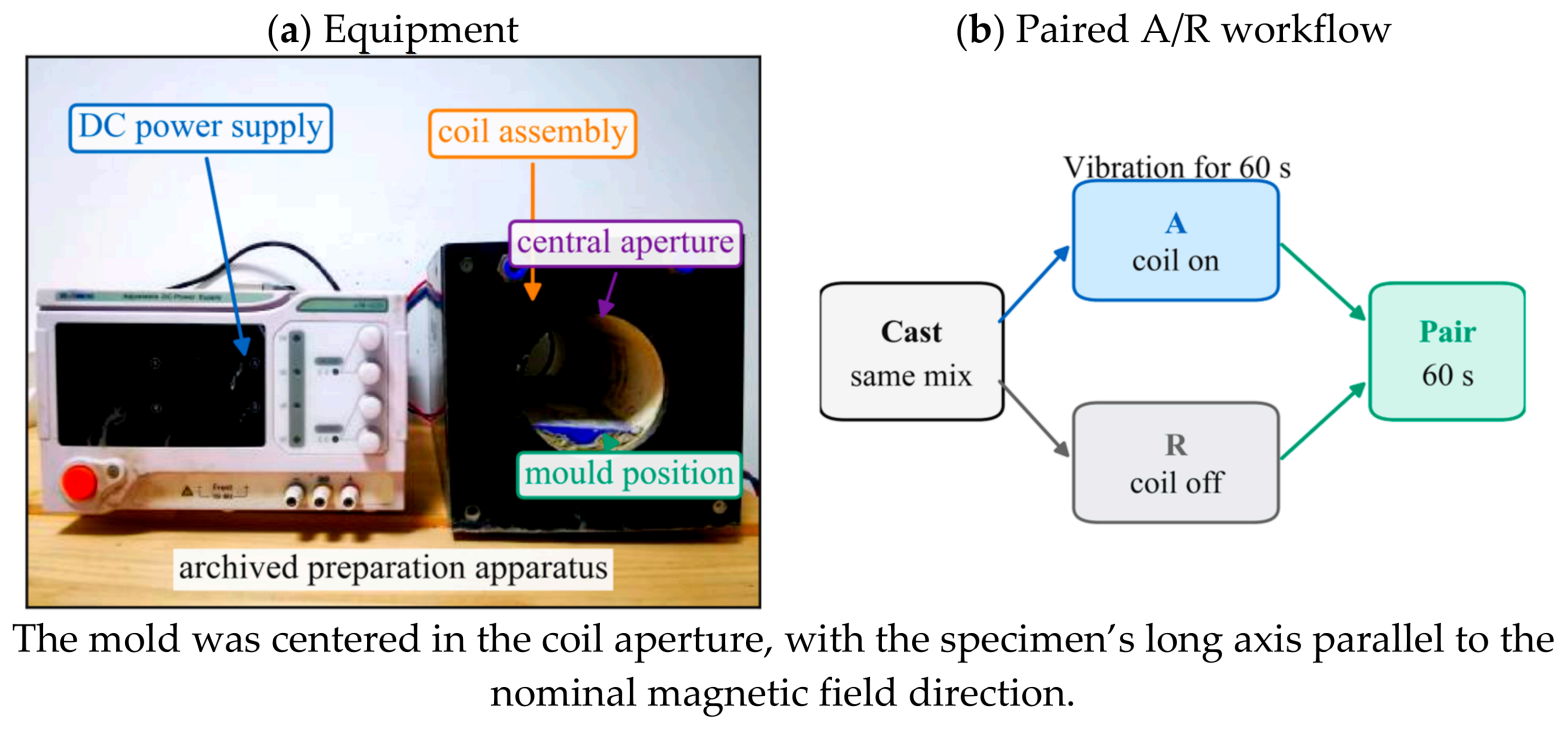
Magnetic field-assisted preparation equipment (**a**), nominal magnetic field direction, and paired A/R workflow (**b**).

The direct-current coil had a central aperture of 100 mm, a conductor diameter of 2.4 mm, 1018 turns, and a resistance of 2.1 Ω. Its rated maximum values were 20 A and 750 G (75 mT). The mold was centered in the aperture with its long axis parallel to the nominal magnetic field. The coil was energized for A and switched off for R during the 60 s vibration stage (Figure 2).

### 2.4. Central Point Flexural Testing

**Figure 3:**
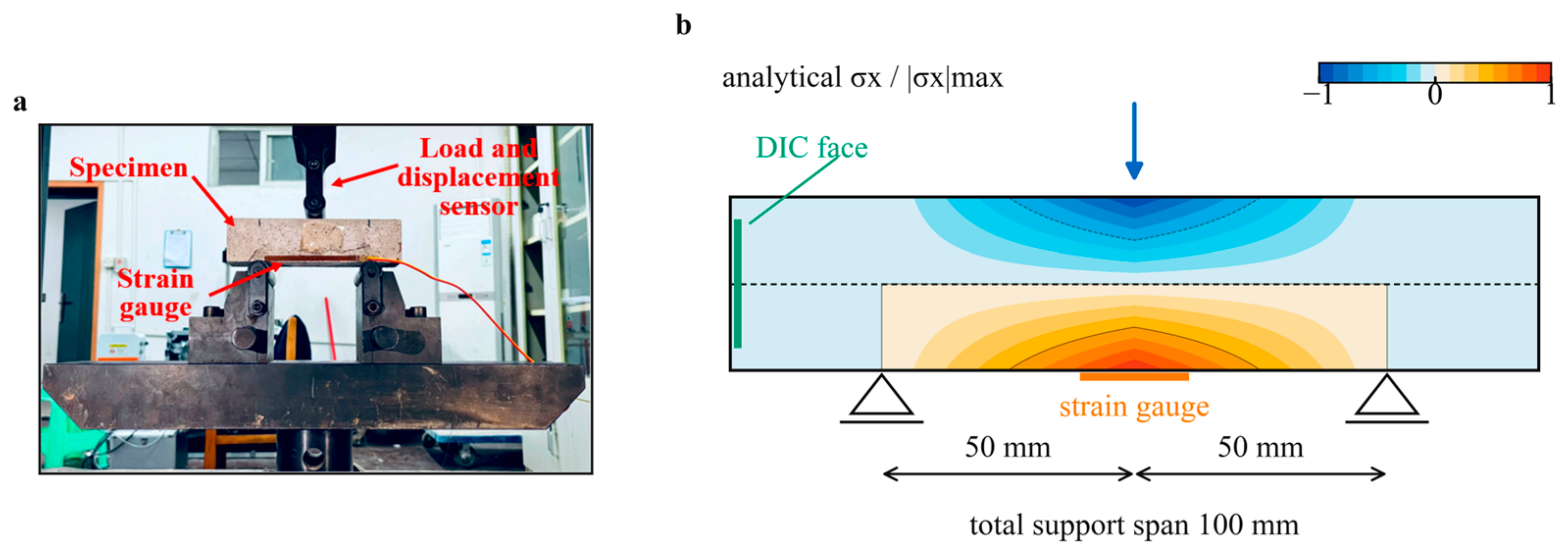
Central point flexural testing setup and schematic stress field. (**a**) Test setup; (**b**) schematic stress field.

At 28 d after casting, central point three-point bending tests were performed using a 100 kN hydraulic testing machine (Figure 3). The span was 100 mm, and the loading point was 50 mm from each support. Each specimen was loaded with the same side face in compression at a displacement rate of 0.1 mm min^−1^. The testing machine recorded load P and displacement δ. A strain gauge on the tension face was used to check the end of the linear segment, and side-face DIC recorded deformation.

Nominal flexural stress was converted from load as follows:

(1) $$f\left(\delta \right)=\frac{3P\left(\delta \right)L}{2bh^{2}}$$

Here, b = h = 40 mm and L = 100 mm; P and stress are expressed in N and MPa, respectively. LOP, peak, and fixed-displacement stresses were all calculated using Equation (1).

### 2.5. Flexural Metrics, Image Analysis, and Statistical Methods

#### 2.5.1. Characteristic Responses and Limit of Proportionality

The limit of proportionality (LOP) was identified at the sustained departure from the initial linear segment of the load–displacement curve, with assistance from the tension-face strain. LOP, peak, and late-stage stresses were converted using Equation (1).

#### 2.5.2. Flexural Energy and Metrics at Common Comparison Displacements

After correction of the initial offset, the reset monotonic portion of each record was retained, duplicate displacements were merged, and linear interpolation was performed at common comparison points while preserving the peak and area. Cumulative work was integrated using the trapezoidal rule:

(2) $$T\left(\delta_{j}\right)=10^{-3}\int_{0}^{\delta_{j}}P\left(\delta \right)d\delta$$

P, δ, and T are expressed in N, mm, and N·m, respectively. Cumulative work at LOP, peak, 3δLOP, and 5.5δLOP was obtained by integrating load with respect to machine displacement. Stage-specific work was defined as follows:

(3) $$W_{\mathrm{pre}}=10^{-3}\int_{0}^{\delta_{\mathrm{peak}}}P\left(\delta \right)d\delta$$

(4) $$W_{\mathrm{early}}=10^{-3}\int_{\delta_{\mathrm{peak}}}^{1.0}P\left(\delta \right)d\delta$$

(5) $$W_{\mathrm{late}}=10^{-3}\int_{1.0}^{3.0}P\left(\delta \right)d\delta$$

Characteristic metrics were the means of three specimens per group. One illustrative curve from each of the 20 fiber conditions was used to calculate fixed-displacement stresses and partitioned work. All fiber-containing curves reached their peaks before 1.0 mm and extended beyond 3.0 mm, while the PC record ended at 0.403 mm. Group means were used for characteristic comparisons, and illustrative curves were used to describe stage-specific trends.

#### 2.5.3. Preparation-Associated Gain and Hybrid Synergy

For a positive response metric X at the same composition, the preparation-associated gain of A relative to R was defined as follows:

(6) $$\Omega_{X}=\frac{X_{A}-X_{R}}{X_{R}}\times 100\%$$

The proportion of CSF in the total fiber volume was defined as follows:

(7) $$x_{\mathrm{CSF}}=\frac{V_{\mathrm{CSF}}}{V_{\mathrm{CSF}}+V_{\mathrm{FSF}}}$$

The x_CSF values were 0.33, 0.50, 0.67, and 0.75. At a total fiber content of 1.60 vol.%, the linear mixture baseline under the same preparation condition was defined as follows:

*X*_ROM_ = (1 − *x*_CSF_)*X*_FSF1.60_ + *x*_CSF_*X*_CSF1.60_ (8)

The endpoints were FSF1.60 and CSF1.60 under the corresponding A or R condition. Hybrid synergy was defined as the percentage departure from this baseline:

(9) $$\Psi_{X}=\frac{X_{\mathrm{hybrid}}-X_{\mathrm{ROM}}}{X_{\mathrm{ROM}}}\times 100\%$$

The departure characterizes the relationship between the hybrid response and the specified linear reference.

#### 2.5.4. Qualitative Imaging and Statistical Interpretation

Two-dimensional DIC used black speckles on a white side-face background, a fixed camera, and independent lighting, with images acquired every 10 s. The Ncorr [49] parameters were a circular subset radius of 20 pixels, a center step of 1 pixel (spacing = 0), a strain-fitting radius of 10 grid intervals, a difference-norm threshold of 10^−6^, and a maximum of 50 iterations. Peak-load frames of selected specimens were used for qualitative comparison of localization and crack paths.

Fracture surfaces were examined for A and R specimens from the FSF0.80, CSF1.60, and 2:1 hybrid groups. SEM used the SE2 signal at 5.00 kV.Mean rankings and percentage differences were evaluated descriptively. The highest values refer to the highest means among the measured conditions. Qualitative images describe morphology, and the orientation, activation, and dispersion parameters were identified from the mechanical responses in Section 4.7.

## 3. Experimental Results and Discussion

### 3.1. Overall Response and Crack Initiation

**Figure 4:**
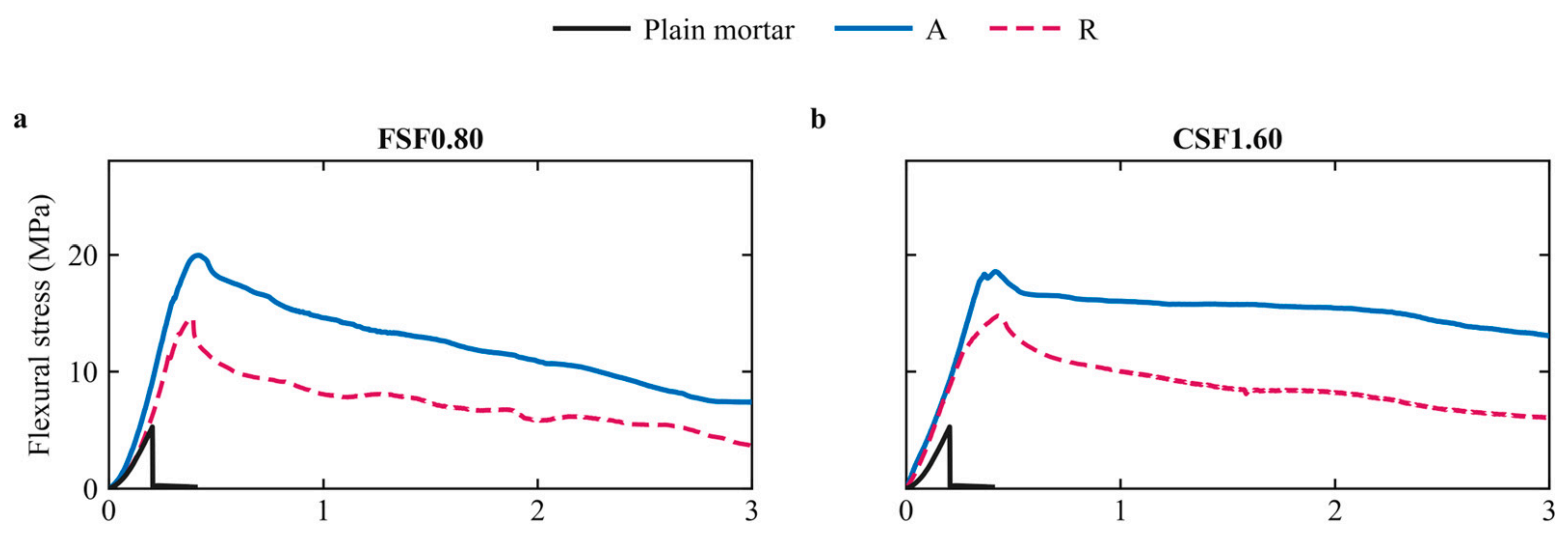
Illustrative flexural responses of single-fiber mortars. The horizontal axis is machine displacement δ (mm). (**a**) FSF0.80; (**b**) CSF1.60.

**Figure 5:**
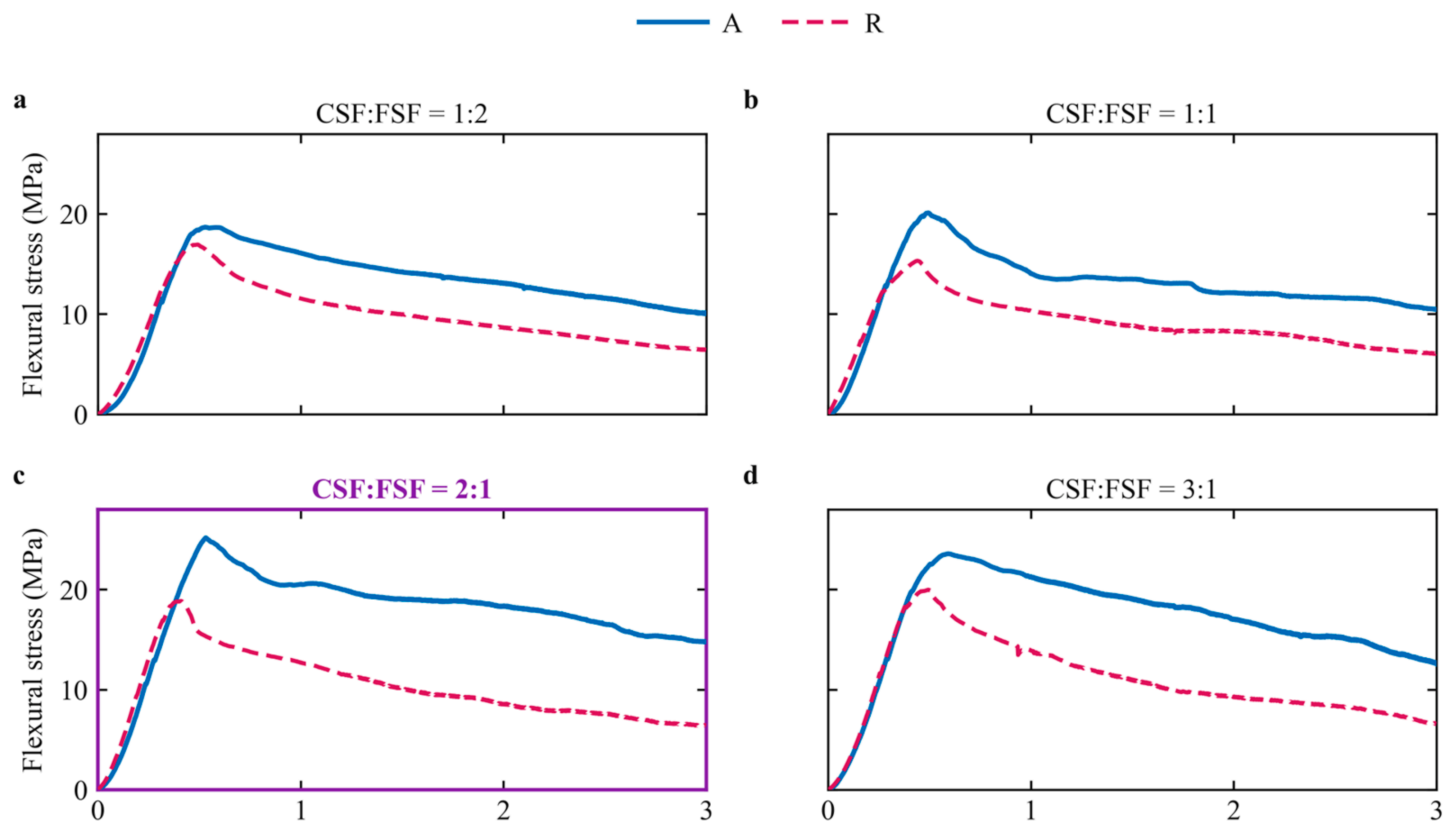
Illustrative flexural responses of hybrid mortars at a total fiber content of 1.60 vol.%. The horizontal axis is machine displacement δ (mm). (**a**) CSF:FSF = 1:2; (**b**) CSF:FSF = 1:1; (**c**) CSF:FSF = 2:1; (**d**) CSF:FSF = 3:1.

PC reached 5.265 MPa at 0.203 mm. The fiber mortars had higher peak stresses, and their curves extended beyond 3.0 mm. FSF0.80 showed a stronger early-stage response, while CSF1.60 maintained higher late-stage resistance (Figure 4). CSF1.07–FSF0.53-A (2:1) achieved the highest mean peak stress in the hybrid series (Figure 5).

The response progressed through three stages: matrix dominance, stress redistribution and fiber activation, and post-peak bridging. These stages were characterized by crack initiation stress, peak stress, late-stage stress, and cumulative work.

### 3.2. Fiber Dosage and Scale Effects

Table 2 summarizes the mean characteristic flexural metrics of three specimens per group for comparisons of dosage, fiber scale, and hybrid composition.

**Figure 6:**
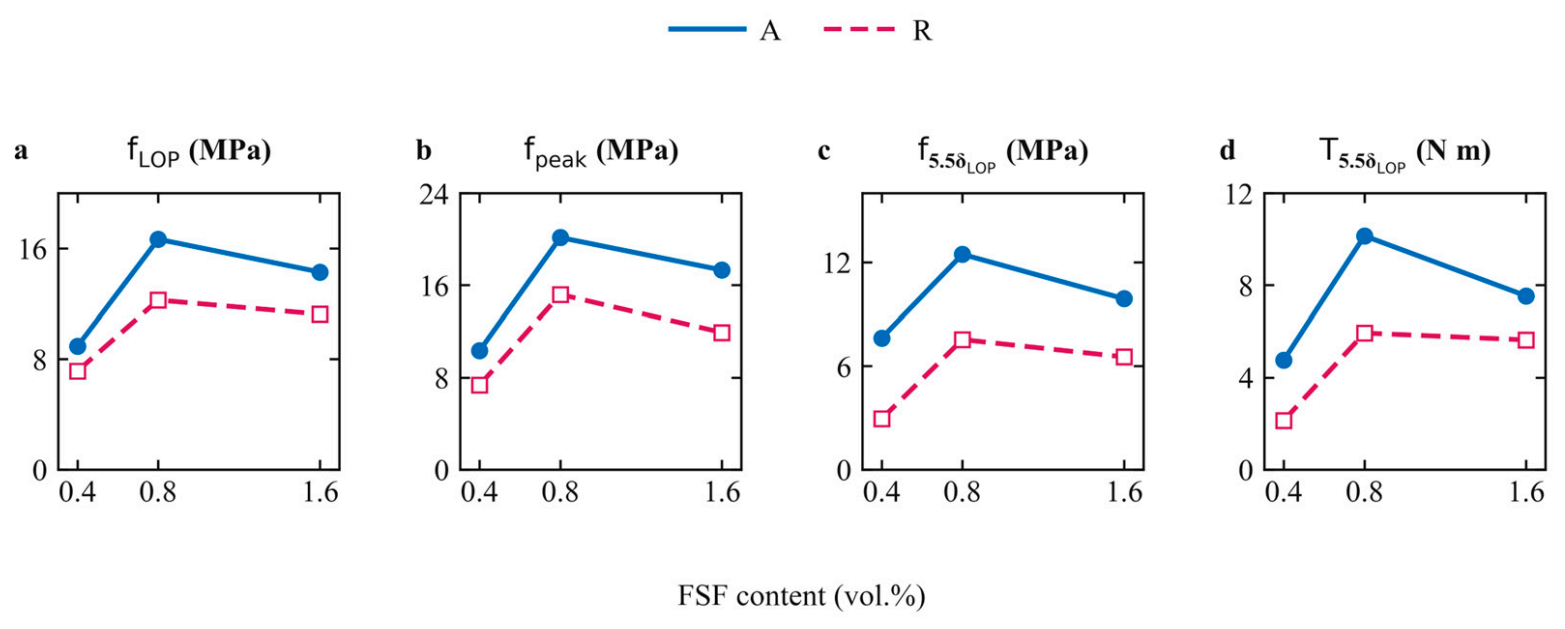
Relationships between FSF content and flexural metrics. (**a**) f_LOP_ (MPa); (**b**) f_peak_ (MPa); (**c**) f_5.5δLOP_ (MPa); (**d**) T_5.5δLOP_ (N m).

At FSF contents of 0.40%, 0.80%, and 1.60%, peak stresses in the A groups were 10.33, 20.14, and 17.31 MPa, respectively, and cumulative energies were 4.75, 10.14, and 7.53 N·m. The 0.80% group had the highest values in both the A and R series (Figure 6).

**Figure 7:**
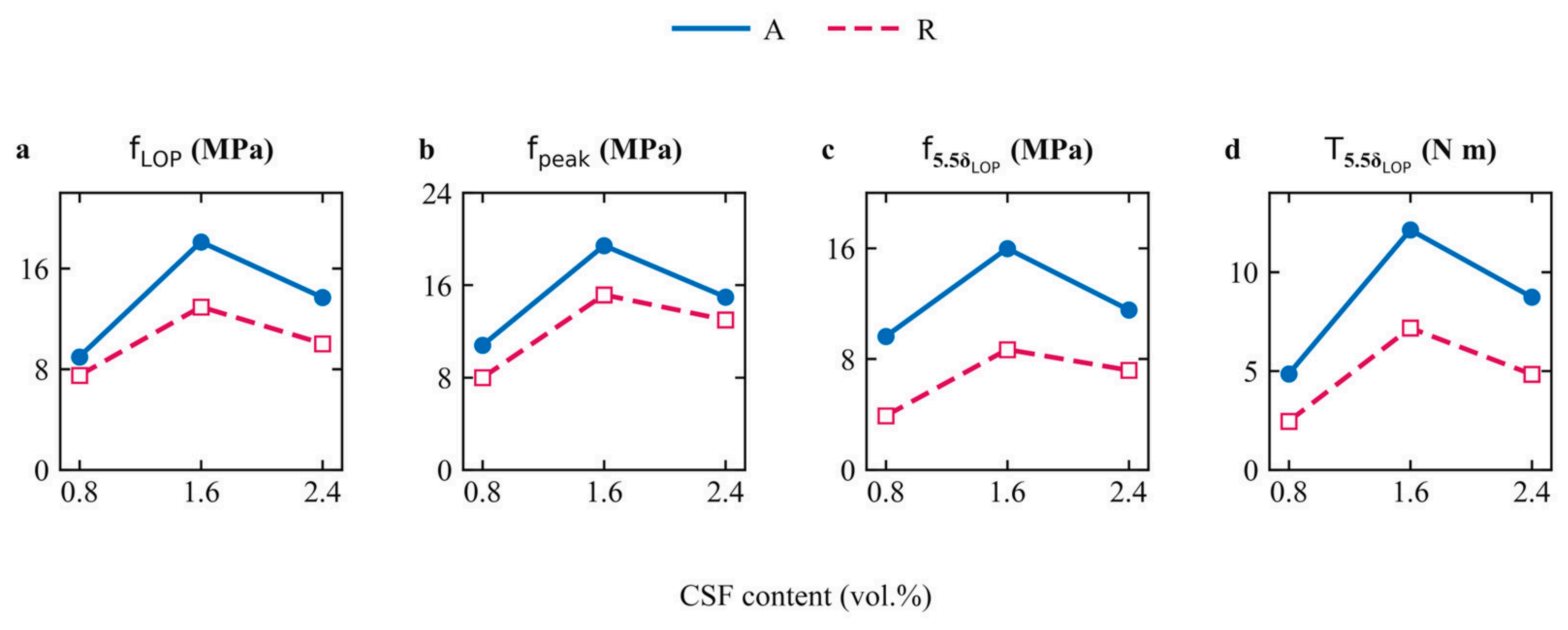
Relationships between CSF content and flexural metrics. (**a**) f_LOP_ (MPa); (**b**) f_peak_ (MPa); (**c**) f_5.5δLOP_ (MPa); (**d**) T_5.5δLOP_ (N m).

At CSF contents of 0.80%, 1.60%, and 2.40%, peak stresses in the A groups were 10.79, 19.43, and 14.97 MPa, respectively, and cumulative energies were 4.85, 12.12, and 8.72 N·m. The 1.60% group had the highest values in both the A and R series (Figure 7).

Under A conditions at the same content of 0.80 vol.%, FSF exceeded CSF in peak stress and cumulative energy by 86.7% and 109.3%, respectively. At the same content of 1.60 vol.%, CSF exceeded FSF by 12.2% and 60.9%, respectively, demonstrating the joint influence of scale and dosage.

### 3.3. Preparation-Associated Gain Across Loading Stages

The A groups had higher mean characteristic stresses than the R groups (Table 2). Peak gains were 15.3–45.3% for single-fiber groups and 18.7–34.9% for hybrid groups; late-stage stress gains were 51.3–159.2% and 42.2–75.3%, respectively, and cumulative energy gains were 33.6–123.1% and 55.3–95.8%. Crack initiation stress gains were 19.7–39.9% and 15.4–23.7%, respectively.

The peak stress, late-stage stress, and cumulative energy of 2:1-A were 26.26 MPa, 18.39 MPa, and 17.06 N·m, respectively, exceeding the corresponding R group by 34.9%, 75.3%, and 95.8%. Relative gains also depend on the R baseline. FSF0.40 had the highest relative energy gain, while FSF0.80 had higher absolute energy.

### 3.4. Hybrid Composition and Departure from the Baseline

**Figure 8:**
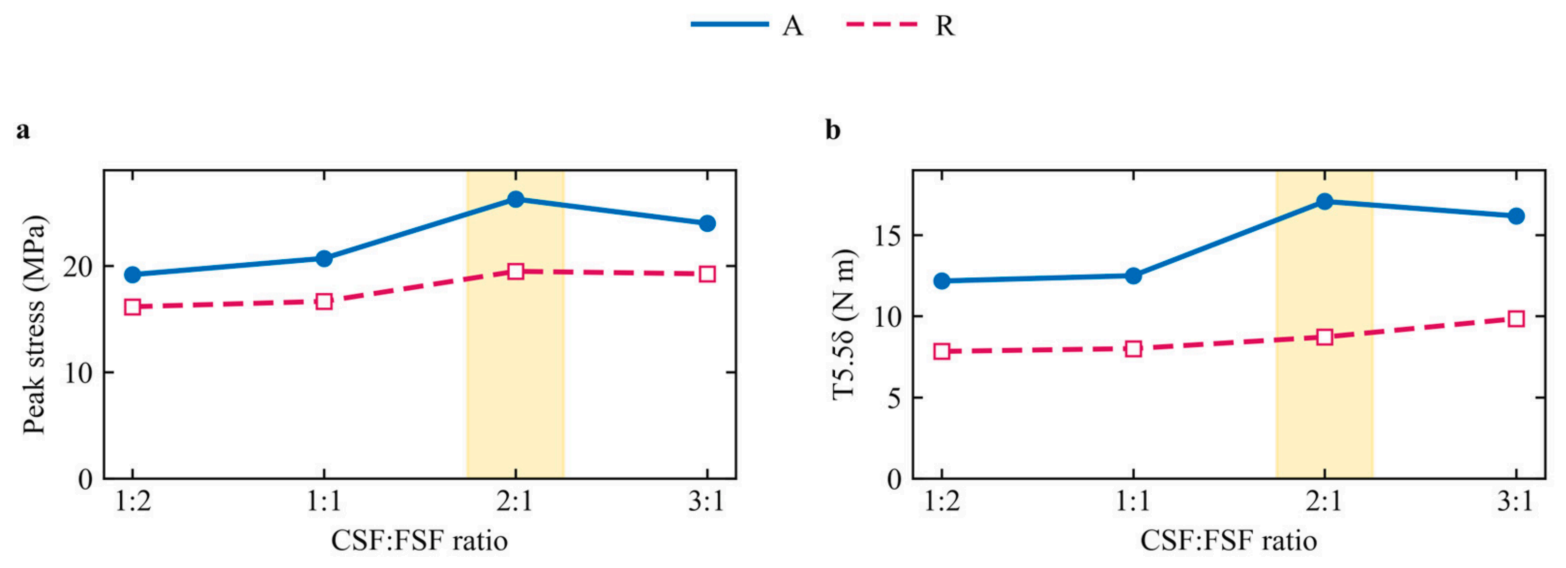
Measured peak stresses and cumulative energies of hybrid mortars. (**a**) Peak stress; (**b**) Cumulative energy.

Among the four hybrid compositions, 2:1-A had the highest mean peak stress and cumulative energy, at 26.26 MPa and 17.06 N·m, respectively (Figure 8); its corresponding crack initiation and late-stage stresses were 19.96 and 18.39 MPa. The highest peak stress and energy groups in the R series were 2:1 and 3:1, respectively, with the latter reaching 9.84 N·m.

The peak stress and cumulative energy of 2:1-A exceeded those of 1:1-A by 26.9% and 36.6%, respectively, and those of 3:1-A by 9.5% and 5.5%. Among the four tested compositions, 2:1-A achieved the highest means for both peak stress and energy.

**Figure 9:**
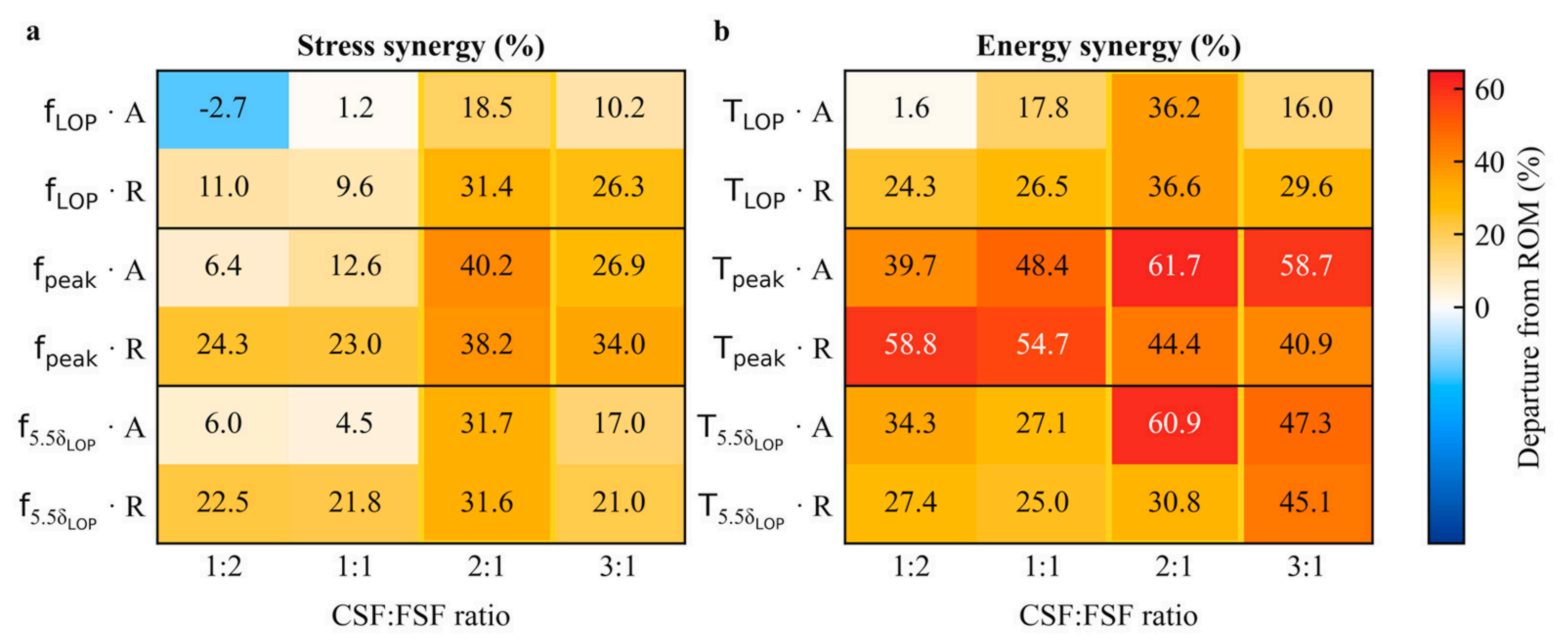
Departures of hybrid responses from rule-of-mixtures baselines under matched preparation conditions. (**a**) Stress synergy; (**b**) Energy synergy.

The peak stress and cumulative energy of each hybrid group exceeded the linear rule-of-mixtures baseline under the corresponding A or R conditions (Figure 9). Peak departures for the four ratios in the A series were 6.4%, 12.6%, 40.2%, and 26.9%, respectively, compared with 24.3%, 23.0%, 38.2%, and 34.0% in the R series. The highest energy departures were 60.9% for 2:1-A and 45.1% for 3:1-R.

### 3.5. Fixed-Displacement Responses and Partitioned Work

**Figure 10:**
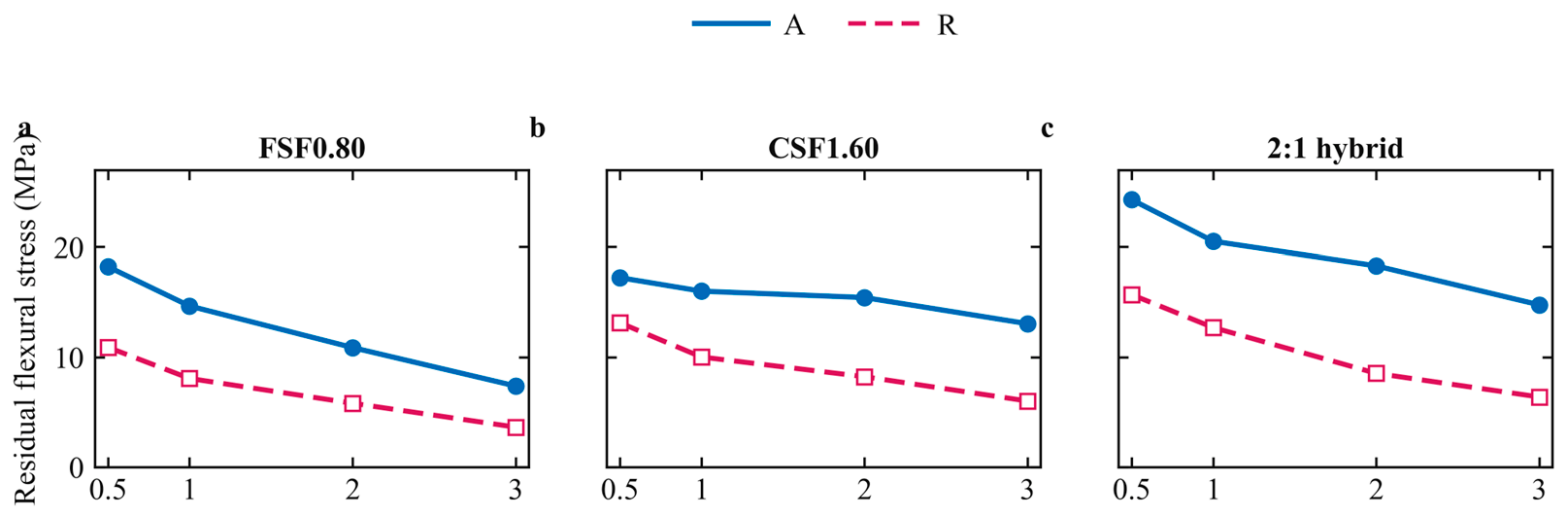
Residual stresses of illustrative curves at fixed machine displacements. The horizontal axis is machine displacement δ (mm). (**a**) FSF0.80, (**b**) CSF1.60, (**c**) 2:1 hybrid.

One illustrative curve per condition was used for fixed-displacement comparisons (Figure 10). At 0.5 mm, the A/R stresses of FSF0.80, CSF1.60, and the 2:1 group were 18.24/10.91, 17.24/13.14, and 24.29/15.70 MPa, respectively; at 3.0 mm, they were 7.39/3.65, 13.06/6.02, and 14.77/6.39 MPa. CSF and the 2:1 group maintained higher late-stage resistance.

The 2:1 group also had the highest illustrative-curve stresses at 1.0 and 2.0 mm, with A/R values of 20.52/12.71 and 18.31/8.54 MPa, respectively.

**Figure 11:**
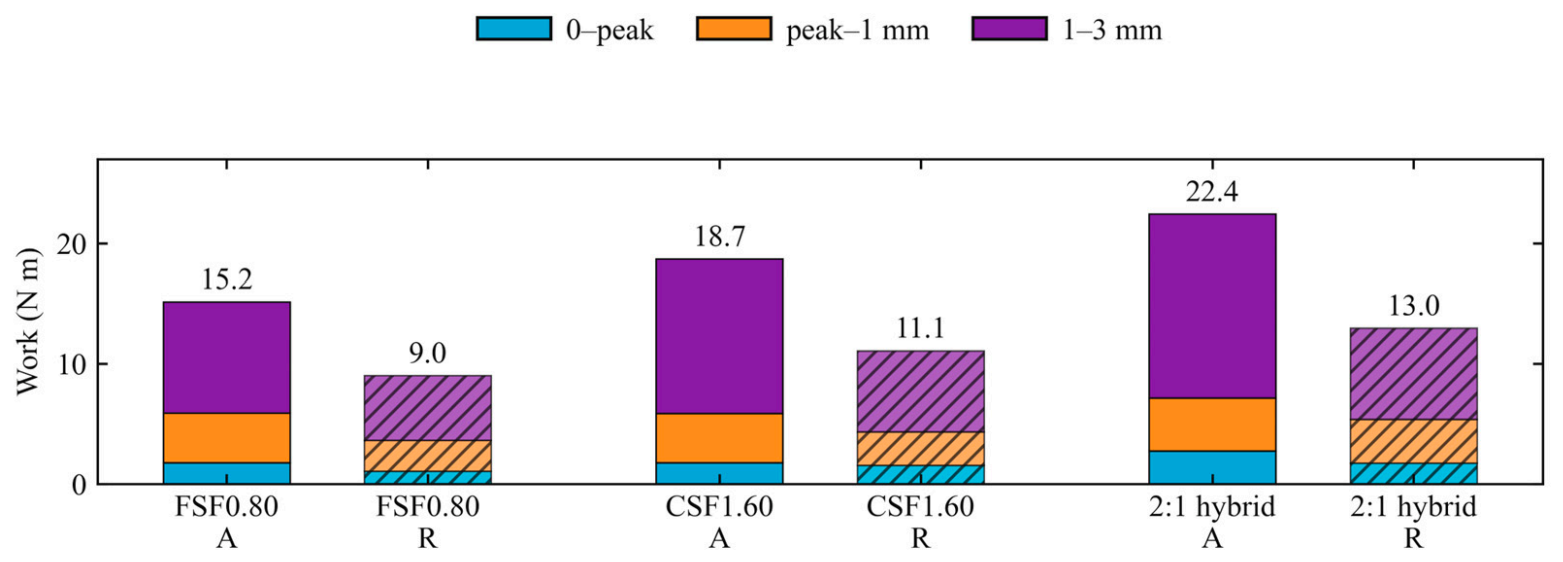
Partitioned work of illustrative flexural responses for single-fiber and hybrid mortars.

Over the 1.0–3.0 mm interval, the partitioned work of the FSF0.80-A, CSF1.60-A, and 2:1-A illustrative curves was 9.25, 12.81, and 15.27 N·m, respectively, exceeding the matched R curves by 72.4%, 90.0%, and 101.2%. These results demonstrate the late-stage gain associated with A preparation (Figure 11).

### 3.6. Crack Fields and Fracture Surface Morphology

**Figure 12:**
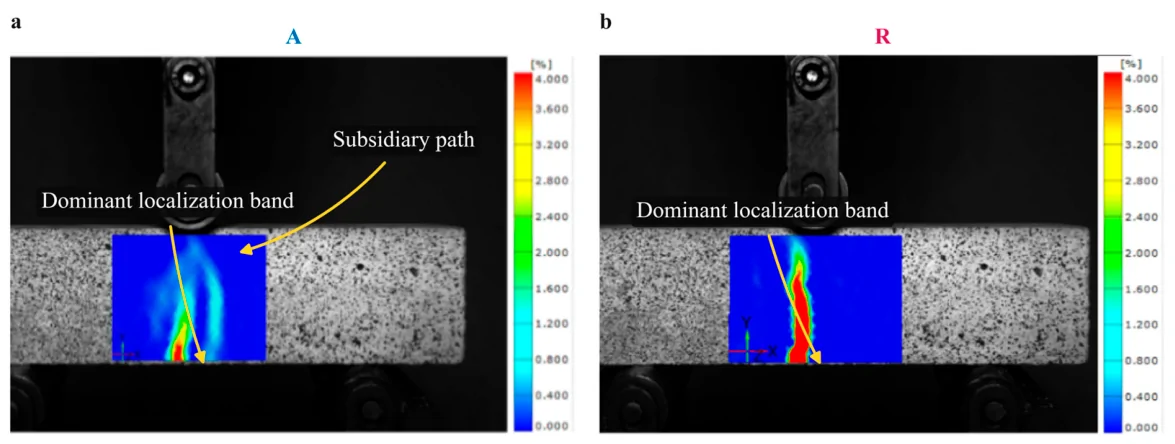
Example DIC localization patterns of FSF0.80 specimens, (**a**) A; (**b**) R.

**Figure 13:**
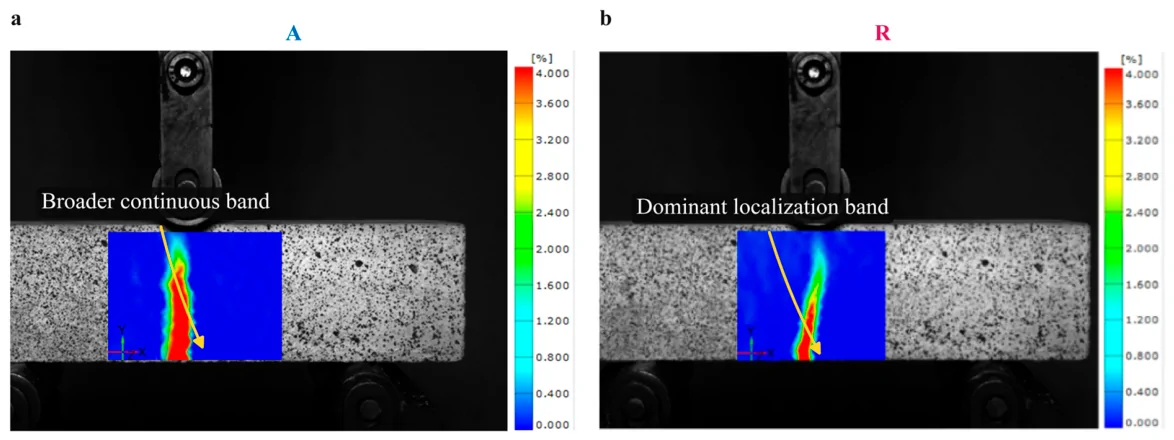
Example DIC localization patterns of CSF1.60 specimens, (**a**) A; (**b**) R.

**Figure 14:**
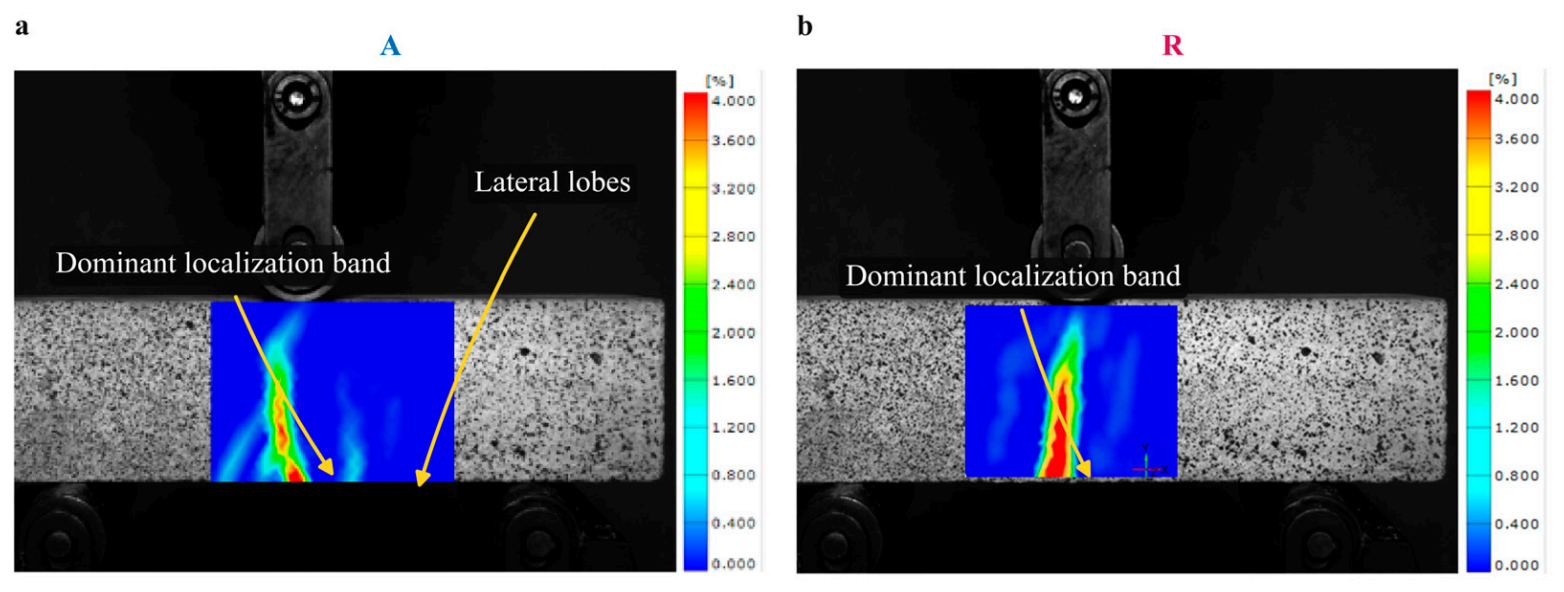
Example DIC localization patterns of 2:1 hybrid specimens, (**a**) A; (**b**) R.

In the DIC examples, FSF0.80-A showed a main band and secondary paths (Figure 12), CSF1.60-A showed a wider continuous band (Figure 13), and 2:1-A showed lobe-shaped regions on both sides of the main band (Figure 14). The R images showed more concentrated main paths, illustrating qualitative differences in localization patterns and crack paths.

**Figure 15:**
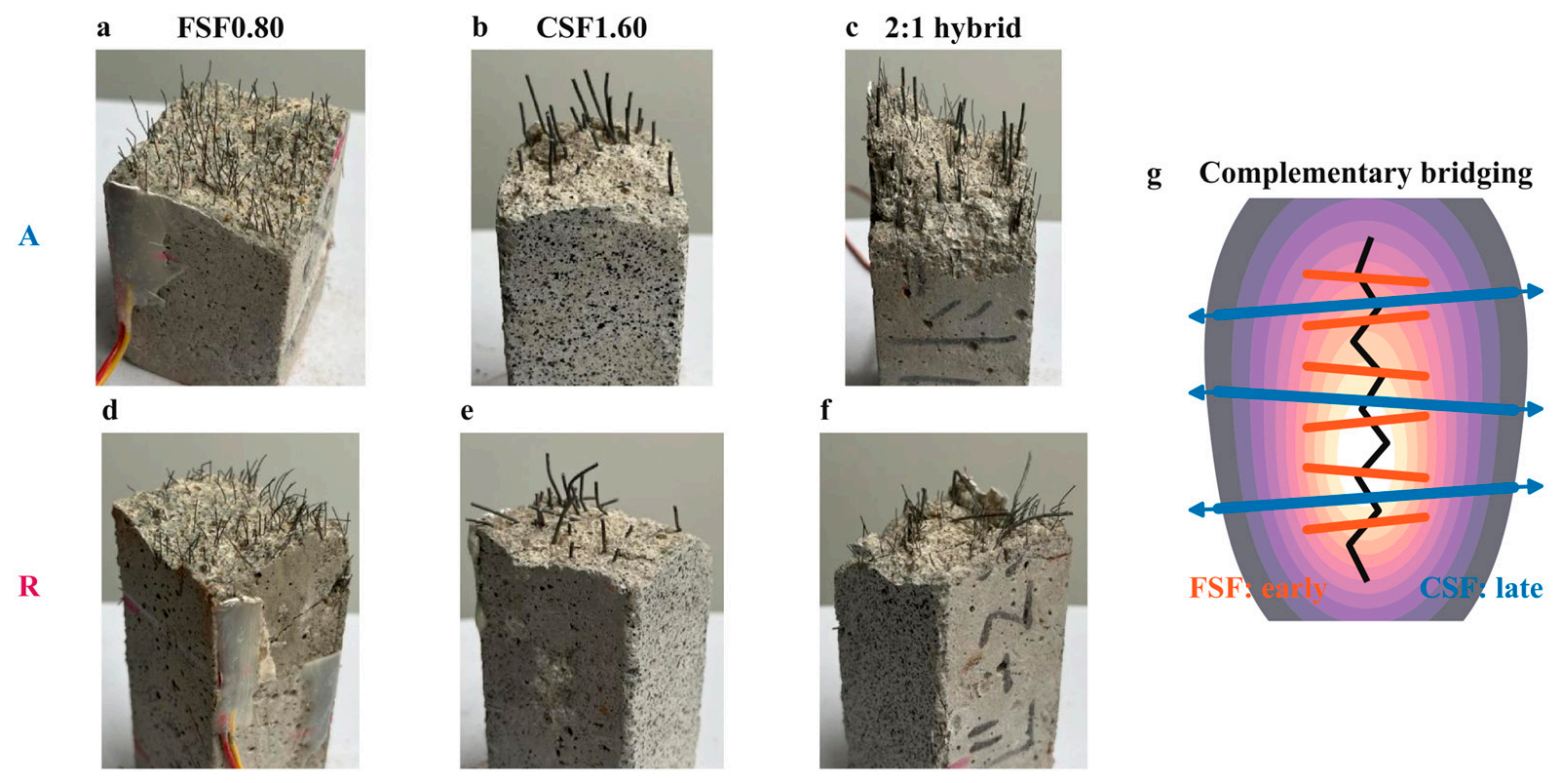
Fracture surface morphology and exposed fibers at two scales. (**a**) FSF0.80-A; (**b**) CSF1.60-A; (**c**) 2:1 hybrid-A; (**d**) FSF0.80-R; (**e**) CSF1.60-R; (**f**) 2:1 hybrid-R; (**g**) Complementary bridging.

Fracture surface photographs showed exposed fibers, attached mortar, and pullout segments. The A fields of view showed exposed fibers approximately normal to the fracture surface, while the hybrid fracture surfaces showed both fine and coarse fibers (Figure 15), providing qualitative observations of fiber arrangement and fracture morphology.

## 4. Multiscale Bridging Model of Orientation and Dispersion

The model successively converts dosage into potential fiber populations, effective crack crossings, single-fiber forces, bridging traction, and sectional moment and then maps these quantities to the nominal stress–machine displacement relationship. Parameters were identified from the mean flexural responses of 12 single-fiber groups to interpret the stage-specific contributions of the two fiber types.

### 4.1. Material Model, Variables, and Assumptions

Fiber type *i* ∈ {*F*, *C*} denotes FSF and CSF, and the preparation condition is *o* ∈ {*A*, *R*}. Volume fractions *V_F_* and *V_C_* determine the total dosage and composition fractions:

*V_t_* = *V_F_* + *V_C_*. (10)

The component fractions are

(11) $$x_{C}=\frac{V_{C}}{V_{t}},$$

*x_F_* = 1 − *x_C_*. (12)

Volume fractions are expressed as decimals, with *V_t_* = 0.016 for the hybrid system. Fiber diameter, length, cross-sectional area, volume, and aspect ratio are denoted by *d_i_*, *L_i_*, *A_i_*, *υ_i_*, and *λ_i_*, respectively, with the following geometric relationships:

(13) $$A_{i}=\frac{\pi d_{i}^{2}}{4},$$

*υ_i_* = *A_i_L_i_*, (14)

Beam width, depth, span, and load are denoted by *b*, *h*, *L_s_*, and *P*, respectively. The second moment of area *I* and section modulus *W_s_* convert moment *M* to nominal stress *f*:

(15) $$I=\frac{bh^{3}}{12},$$

(16) $$W_{s}=\frac{I}{h/2}=\frac{bh^{2}}{6}.$$

The test geometry uses *b* = *h* = 40 mm and *L_s_* = 100 mm, and the nominal fiber lengths are *L_F_* = 16 mm and *L_C_* = 30 mm. The model adopts a single effective dominant crack and straight, smooth fibers, with elastic load transfer, debonding, frictional pullout, and a tensile force cap describing the force response. A and R share interface and dispersion laws, while preparation effects are represented through effective orientation and activation.

### 4.2. Matrix Response and Crack-Hinge Kinematics

*δ* denotes machine displacement; crack opening and net midspan deflection are defined separately. The load–nominal stress relationship is

(17) $$P=\frac{2bh^{2}}{3L_{s}}f.$$

PC reached its peak at 0.203 mm. A smooth reference expression is

(18) $$f_{m}^{\mathrm{sur}}\left(\delta \right)=f_{m,p}{\left(\frac{\delta }{\delta_{m,p}}\right)}^{r_{m}}\exp\left[r_{m}\left(1-\frac{\delta }{\delta_{m,p}}\right)\right],$$

The reference parameters are *f_m_*_,*p*_ = 3.85 MPa, *δ_m_*_,*p*_ = 0.155 mm, and *r_m_* = 12.0. Calculations use piecewise-linear interpolation *f_m_*(*δ*) of the measured PC response, with recorded values over 0 ≤ *δ* ≤ 0.403 mm and zero outside this interval. Matrix load and cumulative beam energy are

(19) $$P_{m}\left(\delta \right)=\frac{2bh^{2}}{3L_{s}}f_{m}\left(\delta \right),$$

(20) $$T_{m}\left(\delta_{j}\right)=10^{-3}\int_{0}^{\delta_{j}}P_{m}\left(\delta \right)d\delta .$$

Here, 10^−3^ converts N·mm to N·m, and *T_m_* is the cumulative beam energy. The effective modulus *E_m_* jointly represents the stiffness of the matrix, beam, and measurement system. The elastic displacement is

(21) $$\delta_{e}=\frac{PL_{s}^{3}}{48E_{m}I},$$

*q* denotes the positive part. The displacement beyond the elastic component forms the crack-hinge component:

*q* = *max*(*q*, 0), (22)

*δ_h_* = *δ* − *δ_e_*. (23)

*z_h_* = *λ_z_h* is the distance from the tension face to the hinge center. Tension-face opening *w_t_*, maximum crack depth *a_e_*, evolving depth *a*(*δ*), and the linear opening field *w*(*y*, *δ*) are

*z_h_* = *λ_z_h*, 0 < *λ_z_* ≤ 1, (24)

(25) $$w_{t}=\frac{4z_{h}}{L_{s}}\delta_{h}.$$

*a_e_* = *min*(*λ_a_h*, *z_e_*). (26)

(27) $$a\left(\delta \right)=a_{0}+\left(a_{e}-a_{0}\right)\left\{1-\exp\left[-{\left(\frac{w_{t}}{w_{a}}\right)}^{n_{a}}\right]\right\}.$$

(28) $$w\left(y,\delta \right)=\left\{\begin{array}{ll} w_{t}\left(1-\frac{y}{a\left(\delta \right)}\right), & a\left(\delta \right)>0,\;0\leq y\leq a\left(\delta \right),\\ 0, & a\left(\delta \right)=0. \end{array}\right.$$

*a_e_* ≤ *z_e_* keeps the lever arm ≥ 0, with baseline values *a*_0_ = 0 and *n_a_* = 1. Load *P*, elastic displacement, and opening are solved jointly; *w_t_*, *a*, *E_m_*, and their parameters are identified from the flexural response. The opening field decreases from the tension face to zero at the effective crack tip.

### 4.3. Fiber Populations, Effective Orientation, and Dispersion

Number density *n_i_* converts fiber volume into the potential population:

(29) $$n_{i}=\frac{V_{i}}{\upsilon_{i}}=\frac{4V_{i}}{\pi d_{i}^{2}L_{i}}.$$

*θ* is the angle between a fiber and the crack-face normal, and *μ* = cos*θ* is defined as follows:

*μ* = cos*θ*, 0 ≤ *μ* ≤ 1. (30)

The normalized power-law density *p_i_*_,*o*_ and its moment $\eta_{i,o}^{\left(r\right)}$ describe effective orientation. The normalization condition, the general moment, and the first two moments are

(31) $$p_{i,o}\left(\mu \right)=\left(\kappa_{i,o}+1\right)\mu^{\kappa_{i,o}},$$

(32) $$\int_{0}^{1}p_{i,o}\left(\mu \right)d\mu =1.$$

(33) $$\eta_{i,o}^{\left(r\right)}=\int_{0}^{1}\mu^{r}p_{i,o}\left(\mu \right)d\mu =\frac{\kappa_{i,o}+1}{\kappa_{i,o}+r+1},$$

(34) $$\eta_{i,o}^{\left(1\right)}=\frac{\kappa_{i,o}+1}{\kappa_{i,o}+2},$$

(35) $$\eta_{i,o}^{\left(2\right)}=\frac{\kappa_{i,o}+1}{\kappa_{i,o}+3}.$$

The identified parameter *κ_i_*_,*o*_ combines geometric orientation, anchorage retention, and activation probability. Crack-crossing density *ρ_i_*_,*o*_ and the orientation density conditioned on crossing $p_{i,o}^{\times }$ are

(36) $$\rho_{i,o}=n_{i}L_{i}\eta_{i,o}^{\left(1\right)}=\frac{4V_{i}}{\pi d_{i}^{2}}\eta_{i,o}^{\left(1\right)}.$$

(37) $$p_{i,o}^{\times }\left(\mu \right)=\frac{\mu p_{i,o}\left(\mu \right)}{\eta_{i,o}^{\left(1\right)}}.$$

The shorter-side embedment length *l* is uniformly distributed over 0 ≤ *l* ≤ *L_i_*/2, independently of orientation, with density

(38) $$g_{i}\left(l\right)=\frac{2}{L_{i}},\;0\leq l\leq \frac{L_{i}}{2},$$

The model uses a positive increment Δ*κ_i_* > 0 to represent the difference in effective orientation and activation between A and R preparation:

*κ_i_*_,*A*_ = *κ_i_*_,*R*_ + Δ*κ_i_*, Δ*κ_i_* > 0, (39)

Separate increments in effective orientation and activation are assigned to the two fiber types. Dosage is normalized by *V*_0_ = 0.01, and dispersion efficiency *ξ_i_* and effective dosage Φ*_i_* jointly describe the population and activation efficiency:

(40) $${\bar{V}}_{i}=\frac{V_{i}}{V_{0}},$$

(41) $$\xi_{i}\left(V_{i}\right)=\exp\left[-c_{i}{\bar{V}}_{i}^{m_{i}}\right].$$

Φ*_i_*(*V_i_*) = *V_i_ξ_i_*(*V_i_*), (42)

The effective crack-crossing density after the dispersion correction is

(43) $$\rho_{i,o}^{\mathrm{eff}}=\rho_{i,o}\xi_{i}\left(V_{i}\right).$$

**Figure 16:**
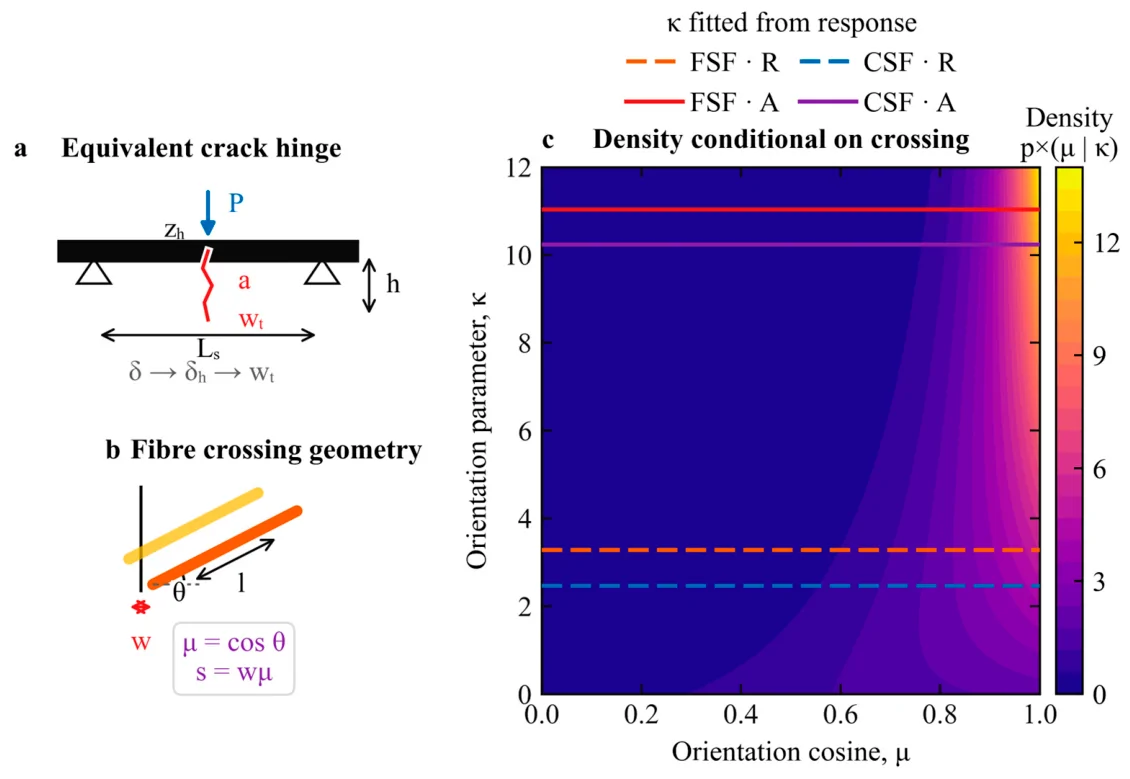
Analytical representations of crack-hinge geometry, slip projection, and effective orientation and activation.

The crossing population uses the first orientation moment, and the force integral further includes the normal projection; the crack projection is included once in the population calculation. Figure 16a,b illustrate the crack hinge and slip mapping, and Figure 16c shows the effective orientation and activation distributions identified from the response.

### 4.4. Single-Fiber Debonding and Pullout

For the shorter-side embedment length *l*, axial stiffness *k_f_*_,*i*_, interfacial tangential stiffness *k_b_*_,*i*_, and the equivalent stiffness in series *k_eq_*_,*i*_ are

(44) $$k_{f,i}\left(l\right)=\frac{E_{i}A_{i}}{l},$$

*k_b_*_,*i*_(*l*) = *K_b_*_,*i*_*πd_i_l*. (45)

(46) $$k_{\mathrm{eq},i}\left(l\right)={\left[\frac{1}{k_{f,i}\left(l\right)}+\frac{1}{k_{b,i}\left(l\right)}\right]}^{-1}.$$

*K_b_*_,*i*_ is the tangential stiffness per unit interfacial area. The interfacial force cap, tensile force cap, critical embedment-length index *l_r_*_,*i*_, and capacity ratio at the maximum embedment length Π*_pr_*_,*i*_ are

*P_b_*_,*i*_(*l*) = *πd_i_τ_i_l*, (47)

*P_u_*_,*i*_ = *A_i_f_u_*_,*i*_. (48)

(49) $$l_{r,i}=\frac{P_{u,i}}{\pi d_{i}\tau_{i}}=\frac{d_{i}f_{u,i}}{4\tau_{i}},$$

(50) $$\Pi_{\mathrm{pr},i}=\frac{P_{b,i}\left(L_{i}/2\right)}{P_{u,i}}=\frac{2\tau_{i}L_{i}}{d_{i}f_{u,i}}.$$

*P_p_*_,*i*_ divides the pullout and rupture modes according to embedment length:

(51) $$M_{i}\left(l\right)=\{\begin{array}{cc} \mathrm{pullout}\;\mathrm{controlled}\;, & P_{p,i}\left(l\right)<P_{u,i},\\ \mathrm{rupture}\;\mathrm{capable}\;\mathrm{or}\;\mathrm{mixed}, & P_{p,i}\left(l\right)\geq P_{u,i}. \end{array}$$

Fibers with shorter embedment transfer force through pullout, while longer embedments provide the conditions for rupture. With *α_i_l* setting the peak slip, the actual peak force, slip at the tensile force cap, and projected slip are

(52) $$s_{p,i}\left(l\right)=\min\left[\frac{P_{b,i}\left(l\right)}{k_{\mathrm{eq},i}\left(l\right)},\alpha_{i}l\right],\;0<\alpha_{i}<1,$$

*P_p_*_,*i*_(*l*) = *k_eq_*_,*i*_(*l*)*s_p_*_,*i*_(*l*). (53)

(54) $$s_{u,i}\left(l\right)=\frac{P_{u,i}}{k_{\mathrm{eq},i}\left(l\right)},$$

*s* = *wμ*. (55)

**Figure 17:**
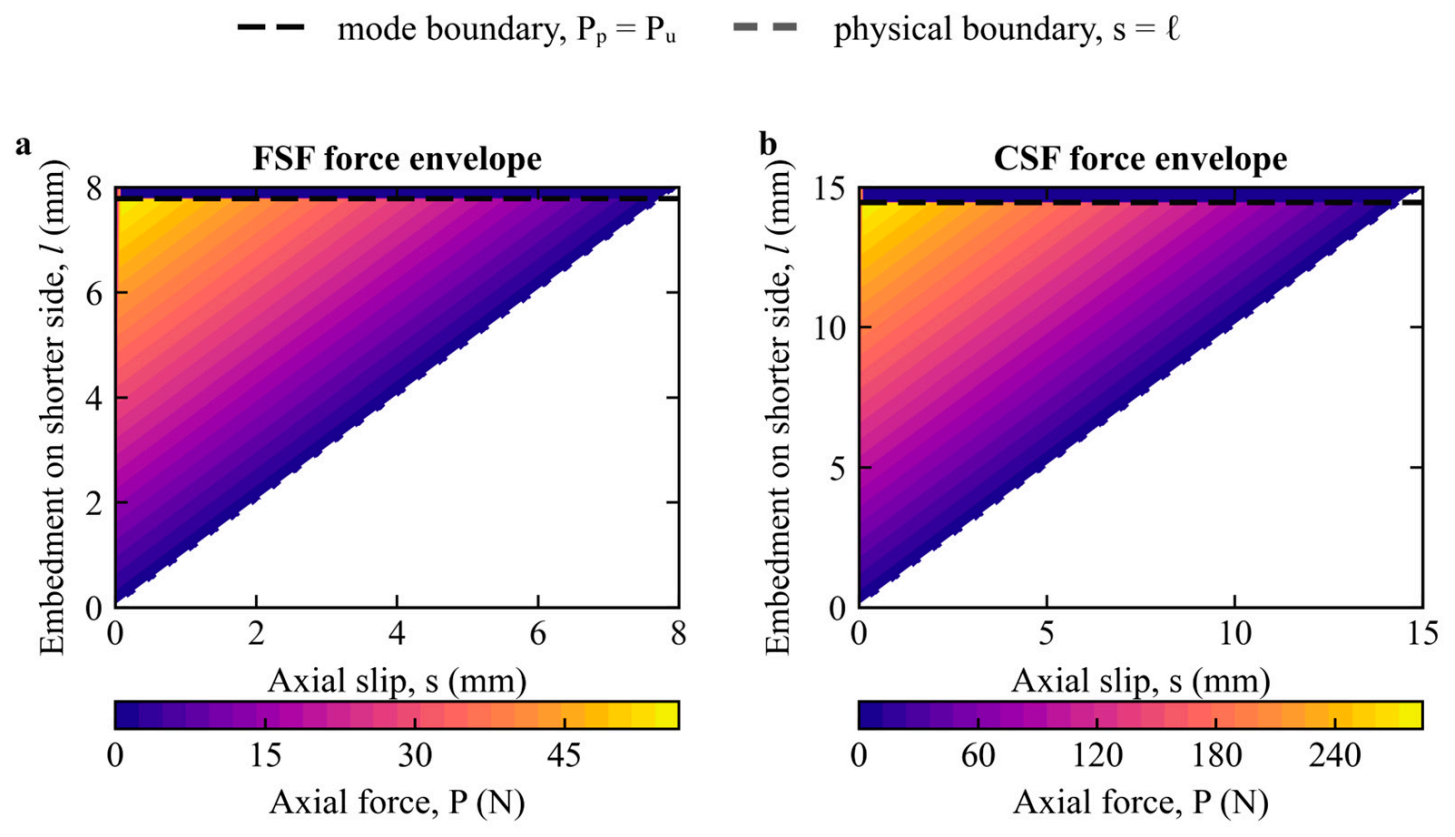
Analytical envelopes of single-fiber force and the pullout and rupture branches.

*P_p_*_,*i*_ = *k_eq_*_,*i*_*s_p_*_,*i*_ represents the actual degree of interface mobilization. The pullout and rupture branches and the normal force are calculated using slip and force projections (Figure 17):

(56) $$P_{i}^{\mathrm{po}}\left(s,l\right)=\{\begin{array}{cc} P_{p,i}\left(l\right)\frac{s}{s_{p,i}\left(l\right)}, & 0\leq s\leq s_{p,i}\left(l\right),\\ P_{p,i}\left(l\right)\frac{l-s}{l-s_{p,i}\left(l\right)}, & s_{p,i}\left(l\right)<s\leq l,\\ 0, & s>l. \end{array}$$

(57) $$P_{i}^{\mathrm{ru}}\left(s,l\right)=\{\begin{array}{cc} k_{\mathrm{eq},i}\left(l\right)s, & 0\leq s\leq s_{u,i}\left(l\right),\\ 0, & s>s_{u,i}\left(l\right). \end{array}$$

(58) $$P_{i}\left(s,l\right)=\{\begin{array}{cc} P_{i}^{\mathrm{po}}\left(s,l\right), & P_{p,i}\left(l\right)<P_{u,i},\\ P_{i}^{\mathrm{ru}}\left(s,l\right), & P_{p,i}\left(l\right)\geq P_{u,i}. \end{array}$$

*Q_i_*(*w*, *μ*, *l*) = *P_i_*(*wμ*, *l*)*μ*. (59)

Single-fiber work *W_f_*_,*i*_ is obtained by integrating axial force with respect to slip. The pullout and idealized rupture expressions are

(60) $$W_{f,i}\left(l\right)=\int_{0}^{\infty }P_{i}\left(s,l\right)ds.$$

(61) $$W_{f,i}^{\mathrm{po}}\left(l\right)=\frac{1}{2}P_{p,i}\left(l\right)l,$$

(62) $$W_{f,i}^{\mathrm{ru}}\left(l\right)=\frac{1}{2}P_{u,i}s_{u,i}\left(l\right)=\frac{P_{u,i}^{2}}{2k_{\mathrm{eq},i}\left(l\right)}.$$

(63) $$W_{f,i}\left(l\right)=\{\begin{array}{cc} W_{f,i}^{\mathrm{po}}\left(l\right), & P_{p,i}\left(l\right)<P_{u,i},\\ W_{f,i}^{\mathrm{ru}}\left(l\right), & P_{p,i}\left(l\right)\geq P_{u,i}. \end{array}$$

The identified values are *τ_F_* = 11.56 MPa, *τ_C_* = 11.41 MPa, *K_b_*_,*F*_ = 2.58 × 10^3^, and *K_b_*_,*C*_ = 2.27 × 10^3^ N·mm^−3^; the capacity ratios are Π*_pr_*_,*F*_ = 1.03 and Π*_pr_*_,*C*_ = 1.04. The series stiffness controls the ascending branch, interface mobilization controls the pullout peak, and the tensile force cap controls the rupture branch. Effective parameters are identified from the beam response, and fiber work is a model-integrated quantity.

### 4.5. Population-Level Bridging Traction and Energy

Integration over effective crack crossings, orientation, and embedment length gives the traction normal to the crack face:

(64) $$\sigma_{b,i,o}\left(w\right)=\rho_{i,o}^{\mathrm{eff}}\int_{0}^{1}\int_{0}^{L_{i}/2}Q_{i}\left(w,\mu ,l\right)p_{i,o}^{\times }\left(\mu \right)g_{i}\left(l\right)dld\mu .$$

FSF and CSF are superposed at the traction level:

*σ_b_*_,*o*_(*w*) = *σ_b_*_,*F*,*o*_(*w*) + *σ_b_*_,*C*,*o*_(*w*). (65)

Bridging work per unit crack area is

(66) $$G_{b,i,o}=\int_{0}^{\infty }\sigma_{b,i,o}\left(w\right)dw.$$

Traction is expressed in N·mm^−2^, bridging work *G_b_* in N·mm^−1^, and beam-test energy in N·m. Bridging work is also obtained by integrating single-fiber work weighted by crossing density:

(67) $$G_{b,i,o}=\rho_{i,o}^{\mathrm{eff}}\int_{0}^{L_{i}/2}W_{f,i}\left(l\right)g_{i}\left(l\right)dl.$$

Population-level bridging work for the two fiber types is

*G_b_*_,*o*_ = *G_b_*_,*F*,*o*_ + *G_b_*_,*C*,*o*_. (68)

An effective opening w^*^ separates early- and late-stage work. The openings corresponding to 50% and 95% of cumulative work and the late-stage work fraction are defined as follows:

(69) $$G_{b,i,o}^{E}=\int_{0}^{w^{*}}\sigma_{b,i,o}\left(w\right)dw,$$

(70) $$G_{b,i,o}^{L}=\int_{w^{*}}^{\infty }\sigma_{b,i,o}\left(w\right)dw.$$

(71) $$\int_{0}^{w_{50,i,o}}\sigma_{b,i,o}\left(w\right)dw=0.50G_{b,i,o},$$

(72) $$\int_{0}^{w_{95,i,o}}\sigma_{b,i,o}\left(w\right)dw=0.95G_{b,i,o}.$$

(73) $$D_{i,o}^{L}=\frac{G_{b,i,o}^{L}}{G_{b,i,o}}.$$

**Figure 18:**
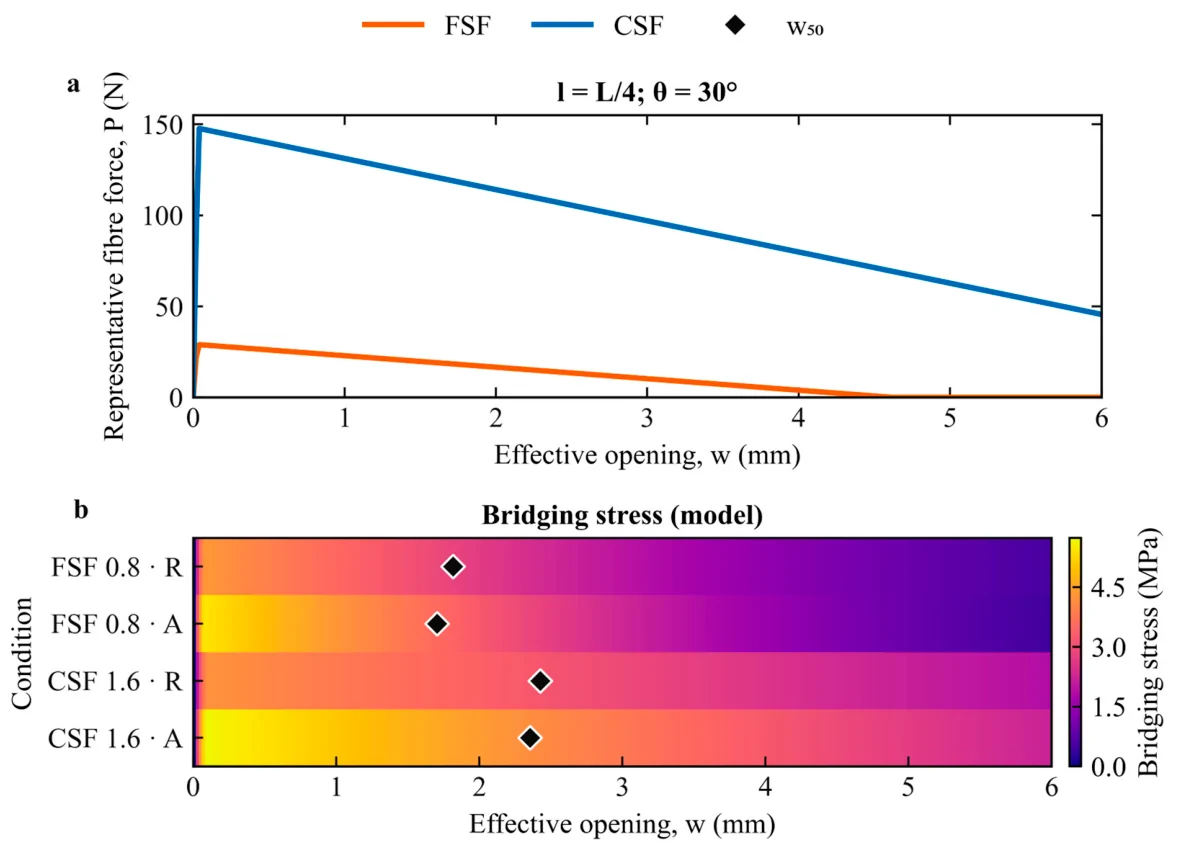
Calculated bridging traction and work at the individual-fiber and single-fiber-system scales.

For FSF/CSF, *w*_50,*F*_/*w*_50,*C*_ is 1.82/2.43 mm under R conditions and 1.71/2.35 mm under A conditions. At representative dosages, *G_b_* is 12.79/17.78 N·mm^−1^ and 15.06/22.90 N·mm^−1^, respectively (Figure 18). Model integration shows that CSF bridging work extends to larger openings.

*w*_50_ and *w*_95_ characterize the central and tail positions of cumulative work, respectively, and *D^L^* is the fraction of work beyond w^*^.

### 4.6. Mapping Bridging Traction to Beam Flexural Response

Integration over the effective crack depth converts traction into its mean value, resultant force, lever arm, moment, and nominal bridging stress:

(74) $${\bar{\sigma }}_{b,o}\left(\delta \right)=\{\begin{array}{cc} \frac{1}{a\left(\delta \right)}\int_{0}^{a\left(\delta \right)}\sigma_{b,o}\left[w\left(y,\delta \right)\right]dy, & a\left(\delta \right)>0,\\ 0, & a\left(\delta \right)=0. \end{array}$$

(75) $$F_{b,o}\left(\delta \right)=b\int_{0}^{a\left(\delta \right)}\sigma_{b,o}\left[w\left(y,\delta \right)\right]dy.$$

*z*(*y*) = *z_e_* − *y*. (76)

(77) $$M_{b,o}\left(\delta \right)=b\int_{0}^{a\left(\delta \right)}\sigma_{b,o}\left[w\left(y,\delta \right)\right]z\left(y\right)dy,$$

(78) $$f_{b,o}\left(\delta \right)=\{\begin{array}{cc} \frac{6M_{b,o}\left(\delta \right)}{bh^{2}}, & a\left(\delta \right)>0,\\ 0, & a\left(\delta \right)=0. \end{array}$$

Adding the matrix and bridging contributions gives the calculated nominal stress and load:

(79) $$f_{o}^{\mathrm{pred}}\left(\delta \right)=f_{m}\left(\delta \right)+f_{b,o}\left(\delta \right).$$

(80) $$P_{o}^{\mathrm{pred}}\left(\delta \right)=\frac{2bh^{2}}{3L_{s}}f_{o}^{\mathrm{pred}}\left(\delta \right).$$

PC interpolation and the bridging contribution are superposed through the effective sectional relationships. The effective opening threshold and corresponding nominal stress are

(81) $$\delta_{\mathrm{LOP}}^{\mathrm{pred}}=\inf\left\{\delta :w_{t}\left(\delta \right)\geq w_{\mathrm{cr}}\right\}.$$

(82) $$f_{\mathrm{LOP}}^{\mathrm{pred}}=f_{o}^{\mathrm{pred}}\left(\delta_{\mathrm{LOP}}^{\mathrm{pred}}\right).$$

*w*_cr_ is the model opening threshold, while experimental LOP is determined as described in Section 2. Beam energy is obtained by integrating calculated load with respect to machine displacement:

(83) $$T^{\mathrm{pred}}\left(\delta_{j}\right)=10^{-3}\int_{0}^{\delta_{j}}P_{o}^{\mathrm{pred}}\left(\delta \right)d\delta .$$

Overall stress corresponds to machine displacement, crack traction corresponds to effective opening, and cumulative beam energy corresponds to the integral of load with respect to machine displacement. Each quantity retains its own coordinate and units throughout the mapping.

Gaussian quadrature generates the traction basis functions. Under-relaxed fixed-point iteration jointly updates load, elastic displacement, opening, crack depth, and moment. Traction is interpolated, and beam energy is integrated using the trapezoidal rule. The calculation units are N, N·mm^−2^, N·mm^−1^, N·mm, and N·m.

The identified kinematic parameters are *λ_z_* = 0.636, *λ_a_* = 0.786, *w_a_* = 0.068 mm, and *w*_cr_ = 0.283 mm, with a baseline lever arm of *z_e_* = 34 mm.

### 4.7. Single-Fiber Parameter Identification and Input Settings

Fixed inputs include material lengths, modulus, beam geometry, analytical diameters, and tensile strength caps. Interface, dispersion, effective orientation, and crack-kinematic parameters are identified from the responses.

Parameter sources and values are listed in Table 3. Lengths and the 200 GPa modulus were taken from the material records. Diameters of 0.20/0.55 mm were adopted with reference to Cui et al. [53] and Lim et al. [54], and tensile strength caps of 1800/1200 MPa were set analytically. The effective system modulus and lever arm were 25 GPa and 34 mm, respectively.

Identification used the mean responses of six R and six A single-fiber groups, including effective LOP stress and displacement, peak response, late-stage stress, and cumulative energy. PC interpolation fixed the matrix response. Positive parameters were represented in scaled or logarithmic coordinates, with search intervals listed in Table 3.

## 5. Model Interpretation of Multiscale Bridging and Morphological Observations

### 5.1. Fiber–Matrix Interface

**Figure 19:**
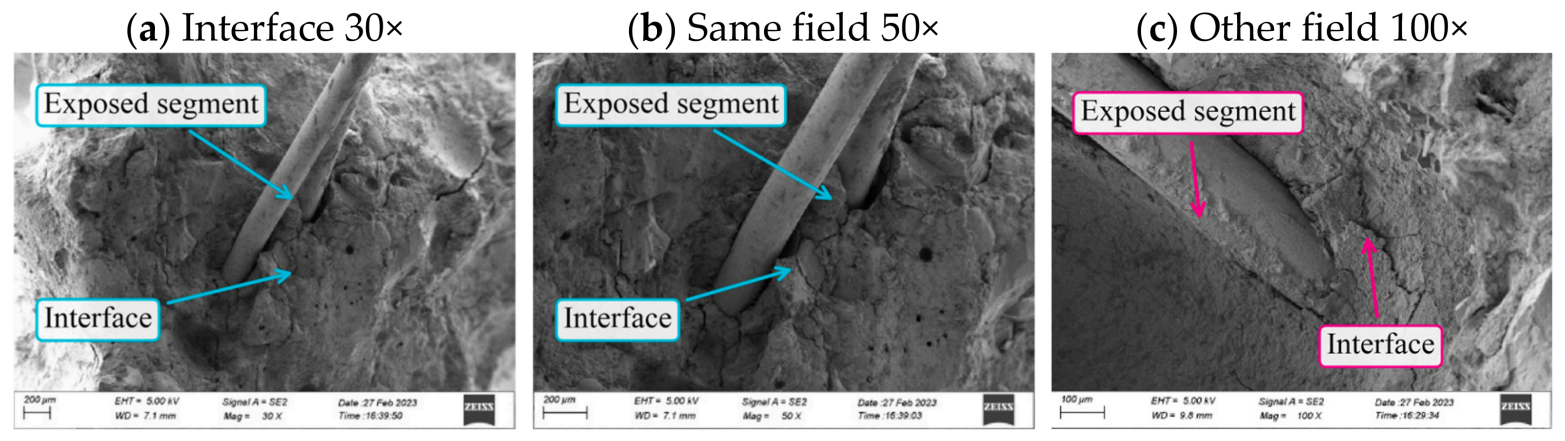
SEM morphology of the fiber–matrix interface. Panels (**a**,**b**) show the same field of view, and panel (**c**) shows a different field of view.

Figure 19a,b show the same field of view at 30× and 50×, respectively, while Figure 19c shows a different field of view at 100×. All images retain their original scale bars and acquisition information. Post-failure morphology shows attached mortar, local interface separation, and exposed fibers.

Flexural curves describe the stage-specific responses, while SEM provides qualitative morphology. Elastic load transfer, debonding, sliding, and pullout are interpreted through the constitutive relationships in Section 4 and connected through traction and sectional equilibrium.

### 5.2. Scale Contributions Across Loading Stages

At 0.80 vol.%, FSF had a higher peak stress than CSF, and the FSF0.80-A DIC image showed secondary localization paths. The model describes the early-stage contribution of fine fibers through *n_F_*, *η_F_*_,*o*_, *ξ_F_*, and local load-transfer stiffness.

The 2:1-A DIC image showed a main band with lobe-shaped regions on both sides, and its fracture surface showed both fine and coarse fibers. The model interprets hybrid bridging during dominant-crack formation through overlapping FSF and CSF traction contributions.

At 3.0 mm, the illustrative stresses of CSF1.60-A and FSF0.80-A were 13.06 and 7.39 MPa, respectively. The cumulative energy of 2:1-A was $T_{5.5\delta_{\mathrm{LOP}}}=17.06$ N·m, and its late-stage illustrative-curve work was *W*_late_ = 15.27 N·m. The model describes late-stage CSF bridging through later characteristic openings and persistent traction, while SEM provides the morphology of exposed fibers.

### 5.3. Preparation, Effective Dispersion, and Hybrid Composition

The larger relative A/R gains occurred in late-stage stress and cumulative energy. The identified relationship *κ_i_*_,*A*_ > *κ_i_*_,*R*_ expresses preparation-associated effective orientation and activation, and fracture surfaces provide qualitative observations of exposed fibers.

The highest tested mean responses of single-fiber groups occurred at 0.80% FSF and 1.60% CSF. The model uses dosage efficiency *ξ_i_*(*V_i_*) to describe the conversion from potential populations to effective crack crossings, with *ξ_i_* identified from the mechanical responses.

At *x_C_* = 0.66875, the 2:1-A combination of 0.53% FSF and 1.07% CSF achieved the highest tested mean peak stress and energy, exceeding the matched A baseline by 40.2% and 60.9%, respectively.

### 5.4. Mixture Selection and Experimental Configuration

Mixture selection combines the preparation and response stages: FSF0.80-A emphasizes the early-to-peak response, while CSF1.60-A maintains late-stage resistance. At a total dosage of 1.60 vol.%, 2:1-A combines higher peak stress and energy; in the R series, the highest means for these two metrics occur at 2:1 and 3:1, respectively.

These mixture comparisons correspond to mortar with a water-to-cement ratio of 0.450, straight smooth fibers of nominal lengths 16/30 mm, 40 × 40 × 160 mm prisms, a 100 mm span, and central point loading at 0.1 mm/min. Preparation used a 60 s vibration workflow, with the A mold centered in the coil aperture and its long axis parallel to the nominal magnetic field direction. This material, preparation, and loading configuration provide the engineering reference for the response comparisons in this study.

## 6. Conclusions

This study established a description of flexural crack bridging through successive stages in hybrid mortar reinforced with steel fibers by combining corrected central point flexural data, work partition at common displacement, qualitative DIC and fracture surface observations, SEM interface evidence, and a multiscale bridging model of orientation and dispersion. Fiber scale governed the stage at which bridging was mobilized; preparation assisted by a magnetic field was associated with higher flexural responses; and the model described the overlap between early and late bridging through effective fiber populations and hybrid composition. The main conclusions are as follows.

(1) Steel fibers changed the response from abrupt fracture to sustained load transfer after the peak. PC reached a peak flexural stress of 5.265 MPa at a machine displacement of 0.203 mm, and its archived trace ended at 0.403 mm, whereas every retained illustrative curve from mortar containing fibers extended beyond 3.0 mm. Both families with a single fiber type showed a rise followed by a decline as dosage increased. FSF0.80-A reached a peak stress of 20.14 MPa and a cumulative energy of 10.14 N m, while CSF1.60-A reached 19.43 MPa and 12.12 N m. The highest reported means among the tested dosages were consequently located at 0.80 vol.% FSF and 1.60 vol.% CSF.
(2) Preparation assisted by a magnetic field increased every A/R response at matched composition, and the gain increased with deformation stage. Gains in peak stress ranged from 15.3% to 45.3% for mixtures with a single fiber type and from 18.7% to 34.9% for hybrid mixtures. At large machine displacement, the corresponding gains in late stress increased to 51.3–159.2% and 42.2–75.3%, while gains in cumulative energy reached 33.6–123.1% and 55.3–95.8%. The principal benefit associated with preparation was sustained crack bridging after peak load.
(3) Hybrid response depended on both composition and deformation stage. CSF1.07–FSF0.53-A, corresponding to a CSF:FSF ratio of 2:1, reached an LOP stress of 19.96 MPa, a peak stress of 26.26 MPa, a late stress of 18.39 MPa, and a cumulative energy of 17.06 N m. Its departures in peak stress and energy from the rule of mixtures baselines with matched preparation were 40.2% and 60.9%, respectively. In the R series, peak stress was highest at 2:1, and cumulative energy was highest at 3:1. Preparation assisted by a magnetic field brought the measured strength and energy maxima together at 2:1.
(4) Responses at common displacement, qualitative DIC localization, fracture surfaces, and SEM morphology support a multiscale bridging sequence with three stages. FSF contributed most effectively during crack initiation and bridging at small openings; both fiber scales overlapped during the formation of the dominant crack; and CSF sustained the response at large openings. At 3.0 mm, illustrative-curve residual stresses were 7.39 MPa for FSF0.80-A, 13.06 MPa for CSF1.60-A, and 14.77 MPa for the 2:1-A hybrid. The 2:1-A curve accumulated 15.27 N m between 1.0 and 3.0 mm, 101.2% above its matched R curve. Attached mortar, interface separation, nearby cracking, and exposed fiber segments in the SEM fields were consistent with elastic transfer, local debonding, frictional sliding, and pullout.
(5) The model of orientation and dispersion connects fiber content, effective crack-crossing populations, single-fiber forces, bridging traction, and beam response. Parameters were identified from the mean responses of six R and six A single-fiber conditions. Effective orientation and activation represent the combined effects of geometric orientation, retained anchorage, and activation probability, while dispersion efficiency describes the conversion of potential fiber populations into effective crack crossings. The single-fiber constitutive laws combine elastic stiffness, interface mobilization, pullout, and tensile strength caps. Population integration and sectional equilibrium link these contributions to the flexural response. The calculated bridging work of CSF persists to larger effective openings, consistent with the sustained late response observed experimentally. The model provides an explanatory framework for the tested mortar, fiber geometry, beam size, and loading configuration.

## Figures and Tables

**Table 1:** Experimental mixture proportions and compositions.

Mixture	CSF (vol.%)	FSF (vol.%)	Total Fiber (vol.%)	n per Condition
FSF0.40	0	0.40	0.40	3
FSF0.80	0	0.80	0.80	3
FSF1.60	0	1.60	1.60	3
CSF0.80	0.80	0	0.80	3
CSF1.60	1.60	0	1.60	3
CSF2.40	2.40	0	2.40	3
CSF0.53–FSF1.07	0.53	1.07	1.60	3
CSF0.80–FSF0.80	0.80	0.80	1.60	3
CSF1.07–FSF0.53	1.07	0.53	1.60	3
CSF1.20–FSF0.40	1.20	0.40	1.60	3

**Table 2:** Mean flexural metrics of the fiber mortar groups (*n* = 3).

Group	f_LOP_ (MPa)	f_peak_ (MPa)	f_5.5δLOP_ (MPa)	T5.5δ (N m)
FSF0.40-A	8.93	10.33	7.62	4.75
FSF0.40-R	7.15	7.35	2.94	2.13
FSF0.80-A	16.66	20.14	12.46	10.14
FSF0.80-R	12.25	15.20	7.53	5.94
FSF1.60-A	14.29	17.31	9.88	7.53
FSF1.60-R	11.24	11.92	6.53	5.64
CSF0.80-A	8.97	10.79	9.63	4.85
CSF0.80-R	7.50	7.99	3.89	2.45
CSF1.60-A	18.10	19.43	15.98	12.12
CSF1.60-R	12.94	15.16	8.68	7.17
CSF2.40-A	13.69	14.97	11.55	8.72
CSF2.40-R	10.02	12.98	7.18	4.82
CSF0.53-FSF1.07-A	15.13	19.16	12.62	12.16
CSF0.53-FSF1.07-R	13.10	16.15	8.87	7.83
CSF0.80-FSF0.80-A	16.39	20.69	13.51	12.49
CSF0.80-FSF0.80-R	13.25	16.65	9.26	8.00
CSF1.07-FSF0.53-A	19.96	26.26	18.39	17.06
CSF1.07-FSF0.53-R	16.26	19.47	10.49	8.71
CSF1.20-FSF0.40-A	18.89	23.98	16.91	16.16
CSF1.20-FSF0.40-R	15.80	19.23	9.85	9.84

**Table 3:** Parameters and fixed inputs of the bridging model.

Parameter	Definition	Unit	Value	Range
*L_F_*	FSF length	mm	16	Fixed
*L_C_*	CSF length	mm	30	Fixed
*d_F_*	FSF analytical diameter	mm	0.2	0.16–0.24
*d_C_*	CSF analytical diameter	mm	0.55	0.44–0.66
*E_F_* = *E_C_*	Steel fiber elastic modulus	GPa	200	Fixed
*f_u_*_,*F*_	FSF analytical tensile strength cap	MPa	1800	1440–2160
*f_u_*_,*C*_	CSF analytical tensile strength cap	MPa	1200	960–1440
*E_m_*	Effective system modulus	GPa	25	20–30
*z_e_*	Effective lever arm	mm	34	30–36
$f_{m,p}^{\mathrm{PC}}$	PC peak flexural stress	MPa	5.265	Measured
$\delta_{m,p}^{\mathrm{PC}}$	PC peak machine displacement	mm	0.203	Measured
$f_{m,p}^{\mathrm{sur}}$	Optional smooth reference amplitude	MPa	3.847	2.63–7.90
$\delta_{m,p}^{\mathrm{sur}}$	Optional smooth reference peak displacement	mm	0.1547	0.05–0.50
$r_{m}^{\mathrm{sur}}$	Optional smooth reference shape parameter	–	12	0.2–12
*λ_z_*	Crack-opening coefficient	–	0.6364	0.35–0.95
*λ_a_*	Maximum crack-depth coefficient	–	0.7861	0.25–0.8
*w_a_*	Opening scale for crack-depth evolution	mm	0.06806	0.05–1.2
*w_cr_*	Model effective LOP threshold	mm	0.2828	0.05–0.6
*τ_F_*	FSF interfacial shear strength	MPa	11.56	0.5–12
*τ_C_*	CSF interfacial shear strength	MPa	11.41	0.5–12
log_10_*K_b_*_,*F*_	Logarithm of FSF interfacial stiffness	–	3.412	1.8–4.8
log_10_*K_b_*_,*C*_	Logarithm of CSF interfacial stiffness	–	3.355	1.8–4.8
*c_F_*	FSF dispersion-loss coefficient	–	0.2062	0.15–2.5
*c_C_*	CSF dispersion-loss coefficient	–	0.1559	0.15–2.5
*m_F_*	FSF dispersion exponent	–	2.845	0.3–8
*m_C_*	CSF dispersion exponent	–	1.759	0.3–8
*κ_F_*_,*R*_	FSF effective orientation activation parameter under R	–	3.283	0–4
Δ*κ_F_*	FSF orientation increment from R to A	–	7.756	0.05–8
*κ_C_*_,*R*_	CSF effective orientation activation parameter under R	–	2.465	0–4
Δ*κ_C_*	CSF orientation increment from R to A	–	7.773	0.05–8
*α_F_*	FSF peak-slip cap	–	0.12	0.08–0.16
*α_C_*	CSF peak-slip cap	–	0.1	0.07–0.13
*w* ^*^	Boundary between early- and late-stage bridging	mm	0.5	0.40–0.60

## Data Availability

The original contributions presented in this study are included in the article. Further inquiries can be directed to the corresponding author.
